# Bone marrow niche-derived extracellular matrix-degrading enzymes influence the progression of B-cell acute lymphoblastic leukemia

**DOI:** 10.1038/s41375-019-0674-7

**Published:** 2020-01-09

**Authors:** Divij Verma, Costanza Zanetti, Parimala Sonika Godavarthy, Rahul Kumar, Valentina R. Minciacchi, Jakob Pfeiffer, Markus Metzler, Sylvain Lefort, Véronique Maguer-Satta, Franck E. Nicolini, Barbara Burroni, Michaela Fontenay, Daniela S. Krause

**Affiliations:** 1 Georg-Speyer-Haus, Institute for Tumor Biology and Experimental Therapy, 60596 Frankfurt am Main, Germany; 2 Department of Pediatrics and Adolescent Medicine, University Hospital Erlangen, Erlangen, Germany; 3 CRCL, Inserm U1052-CNRS UMR5286, Centre Léon Bérard, Lyon, France; 4 Department of Hematology and INSERM U 1052, CRCL, Centre Léon Bérard, 69373 Lyon Cedex, France; 5 Pathology Department, Groupe Hospitalier Cochin, APHP, Paris Descartes University, Paris, France; 6 Laboratory of Hematology Hôpital Cochin, Université Paris Descartes, 75014 Paris, France; 7 Faculty of Medicine, Johann Wolfgang Goethe University, Frankfurt, Germany; 8 Frankfurt Cancer Institute (FCI), Goethe University, Frankfurt am Main, Germany; 9 German Cancer Consortium (DKTK), Heidelberg, Germany; 10 German Cancer Research Center (DKFZ), Heidelberg, Germany

**Keywords:** Cancer microenvironment, Preclinical research

## Abstract

Specific and reciprocal interactions with the bone marrow microenvironment (BMM) govern the course of hematological malignancies. Matrix metalloproteinase-9 (MMP-9), secreted by leukemia cells, facilitates tumor progression via remodeling of the extracellular matrix (ECM) of the BMM. Hypothesizing that leukemias may instruct the BMM to degrade the ECM, we show, that MMP-9-deficiency in the BMM prolongs survival of mice with BCR-ABL1-induced B-cell acute lymphoblastic leukemia (B-ALL) compared with controls and reduces leukemia-initiating cells. MMP-9-deficiency in the BMM leads to reduced degradation of proteins of the ECM and reduced invasion of B-ALL. Using various in vivo and in vitro assays, as well as recipient mice deficient for the receptor for tumor necrosis factor (TNF) α (TNFR1) we demonstrate that B-ALL cells induce MMP-9-expression in mesenchymal stem cells (MSC) and possibly other cells of the BMM via a release of TNFα. MMP-9-expression in MSC is mediated by activation of nuclear factor kappa B (NF-κB) downstream of TNFR1. Consistently, knockdown of TNF-α in B-ALL-initiating cells or pharmacological inhibition of MMP-9 led to significant prolongation of survival in mice with B-ALL. In summary, leukemia cell-derived *Tnf*α induced MMP-9-expression by the BMM promoting B-ALL progression. Inhibition of MMP-9 may act as an adjunct to existing therapies.

## Introduction

Hematopoietic stem cells (HSC) reside in the bone marrow (BM) microenvironment (BMM) which is composed of various cell types such as mesenchymal stem cells (MSC) and others, but also the extracellular matrix (ECM), cytokines, and other factors. Similarly, hematological malignancies are influenced by their BMM in their progression [1] and, conversely, they remodel the BMM suppressing normal hematopoiesis while supporting their own growth [2, 3]. Remodeling of the BMM protects leukemia cells from chemotherapy- or tyrosine kinase inhibitor-induced cell death [4, 5] and inhibition of the remodeling process may increase sensitivity to chemotherapy [5, 6].

The ECM of the BMM consists of collagens, proteoglycans, and glycoproteins and is involved in cell adhesion, binding of growth factors, and other functions [7]. ECM proteins such as tenascin-C [8], osteopontin [9], collagen IX α1 [10], or Del-1 [11] influence hematopoiesis, for instance via direct support of hematopoietic cell growth, regulation of apoptosis, alteration of the bone network, or promotion of HSC proliferation and differentiation, respectively. In leukemia, periostin promotes the growth of B-cell acute lymphoblastic leukemia (B-ALL) [12], while inhibition of osteopontin increases B-ALL burden [5].

BMM-derived proteases such as CD26 [13] or matrix metalloproteinases (MMP) play a role in HSC mobilization [14], while heparanase contributes to HSC retention [15]. Heparanase, cleaving heparan sulfate, and MMPs, which belong to the family of zinc-dependent endopeptidases, are involved in remodeling of the ECM. In cancer, MMPs may be produced by tumor [16] or stromal cells [17], where they degrade the ECM facilitating cancer progression. One such MMP is matrix metalloproteinase-9 (MMP-9) or gelatinase B [14], produced by macrophages, neutrophils [18], fibroblasts [19], MSC [20, 21], and other cells. Its expression may be increased in cancer cells of solid tumors, where it may correlate with metastasis [22], while in chronic lymphocytic leukemia (CLL) leukemia-cell specific, increased expression of MMP-9 contributes to disease progression [23]. Given the published data on tumor cell-specific secretion of MMP-9, we hypothesized that leukemia cells in B-ALL, the most common cancer in children, may remodel the BMM via the production of cytokines or other factors leading to increased expression of MMP-9 by cells of the BMM and, thereby, increased invasiveness of leukemia cells.

Indeed, in this report we show that tumor necrosis factor (*Tnf*)α, secreted by B-ALL cells, leads to increased expression of MMP-9 in MSC via activation of the *Tnf*α receptor (TNFR)1-nuclear factor kappa-light-chain-enhancer of activated B cells nuclear factor kappa B (NF-κB) pathway and increased invasiveness of B-ALL cells. Our results further suggest that MMP-9 inhibition may represent a feasible adjunct treatment strategy in B-ALL, where leukemia relapse and progression remain major concerns.

## Materials and methods

### Mice

C57/BL6 and MMP-9 knockout (KO) mice (on a C57/BL6 background) were purchased from Charles River Laboratories (Sulzfeld, Germany). TNFR1-deficient mice were a kind gift from Prof. Florian Greten, Georg-Speyer-Haus. Heparanase KO mice were obtained from the European Conditional Mouse Mutagenesis Program. All murine studies were approved by the local animal care committee (Regierungspräsidium Darmstadt).

### Statistical analysis

Statistical significance between different treatment groups was assessed by Student’s *t* test using Prism Version 6 software (GraphPad, La Jolla, CA). When multiple hypotheses were tested, one-way ANOVA and a Tukey Test as post hoc test were used. Differences in survival were assessed by Kaplan–Meier nonparametric tests (Log-rank or Wilcoxon tests). Data were presented as mean ± s.e.m, and differences were considered significant when *P* values ≤ 0.05.

## Results

### Deficiency of MMP-9 in the BMM prolongs the survival of mice with B-ALL

**Fig. 1:**
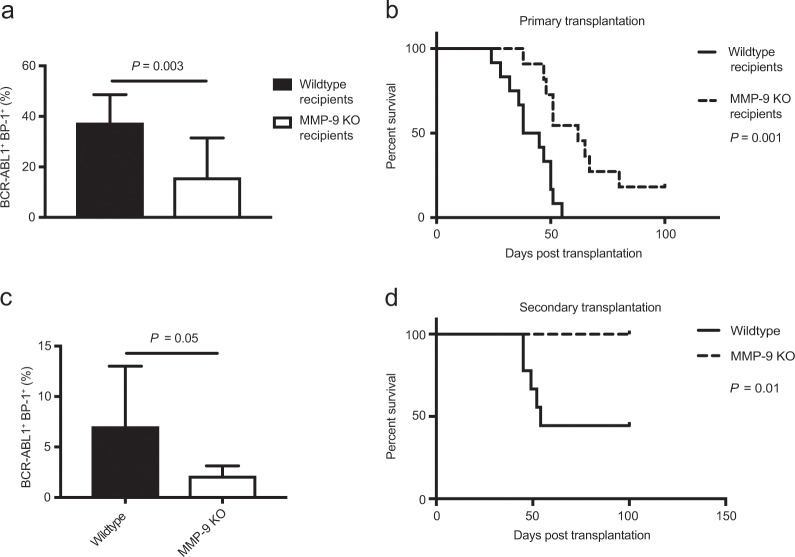
Deficiency of MMP-9 in the BMM prolongs survival of mice with B-ALL. **a** Percentage of GFP^+^ (BCR-ABL1^+^) BP-1^+^ cells in peripheral blood of wild type (black) or MMP-9 KO (white) recipient mice on day 25 of transplantation of BCR-ABL1^+^ transduced bone marrow in the B-ALL model (*P* = 0.003; *t* test, *n* = 10). **b** Kaplan–Meier-style survival curve of wild type (solid line) or MMP-9 KO (dashed line) recipient mice transplanted with 1 × 10^6^ BCR-ABL1-transduced donor bone marrow cells (*P* = 0.001, Log-rank test, *n* = 12). **c** Percentage of GFP^+^ (BCR-ABL1^+^) BP-1^+^ cells in peripheral blood of wild type secondary recipients of BCR-ABL1^+^ bone marrow transplanted from wild type (black) or MMP-9 KO (white) primary mice with established B-ALL on day 18 after transplantation (*P* = 0.05; *t* test, *n* = 8). **d** Kaplan–Meier-style survival curve of wild type secondary recipients of unsorted BCR-ABL1^+^ bone marrow from wild type (solid line) or MMP-9 KO (dashed line) primary mice with established B-ALL (*P* = 0.01, Log-rank test, *n* = 9).

As a role of MMP-9 (and the ECM-degrading enzyme heparanase), derived from the BMM, had not previously been implicated in B-ALL, we transplanted wild type BM transduced with retrovirus expressing the oncogene *BCR-ABL1*, which is associated with 3% of pediatric B-ALL and 25% of adult B-ALL, into heparanase- or MMP-9-deficient or control recipient mice. We observed no significant differences in BCR-ABL1^+^ (GFP^+^) BP-1^+^ pre-B cells in the peripheral blood (Fig. S1A and Table S1) or in the survival of heparanase-deficient compared with wild type recipient mice (Fig. S1B). In contrast, a significantly reduced BCR-ABL1^+^ (GFP^+^) BP-1^+^ leukemia load (*P* = 0.003, Fig. 1a), as well as prolonged survival (*P* = 0.001, Fig. 1b) was observed in MMP-9-deficient mice with B-ALL. In contrast, deficiency of MMP-9 in B-ALL-initiating cells did not alter survival in wild type recipient mice compared with controls (Fig. S1C). No major abnormalities were observed in peripheral blood, BM or spleen (Figs. S2A–E), and percentages of Lin^−^ c-Kit^+^ Sca-1^+^ (LKS) and LKS CD150^+^ CD48^−^ (SLAM) cells (Fig. S2F) in BM or spleen and colony formation (Fig. S2G) did not differ between wild type and MMP-9 KO mice.

We transplanted total BM from wild type versus MMP-9-deficient recipient mice with established B-ALL into wild type secondary recipient mice to assess the self-renewal capacity of leukemia-initiating cells (LIC). This revealed a significant reduction of leukemia load (*P* = 0.05, Fig. 1c), as well as significant prolongation of survival of secondary recipients transplanted with BM from MMP-9-deficient mice with B-ALL (*P* = 0.01, Fig. 1d). In summary, these results suggest that BMM-derived MMP-9 contributes to B-ALL progression, at least partly by reduction of LIC.

### MMP-9-deficiency in the BMM does not alter the homing capacity, cell cycle, or apoptosis of BCR-ABL1^+^ B-ALL cells

Testing the reason for prolonged survival of MMP-9-deficient mice with B-ALL, we did not observe any significant differences in the homing efficiency of GFP^+^ (BCR-ABL1^+^) BP-1^+^ LIC, which represent the LIC in this model (DSK, unpublished data), to a wild type or MMP-9-deficient BMM (Fig. S3A). Analysis of cell cycle (Fig. S3B) or apoptosis (Fig. S3C) of B-ALL cells from an MMP-9-deficient versus a wild type BMM did not reveal significant differences, suggesting that the observed differences in survival of wild type versus MMP-9 KO mice were not due to alterations in homing, cell cycle, or apoptosis of leukemia cells [1].

### MMP-9 deficiency leads to higher levels of extracellular matrix proteins in the BMM

**Fig. 2:**
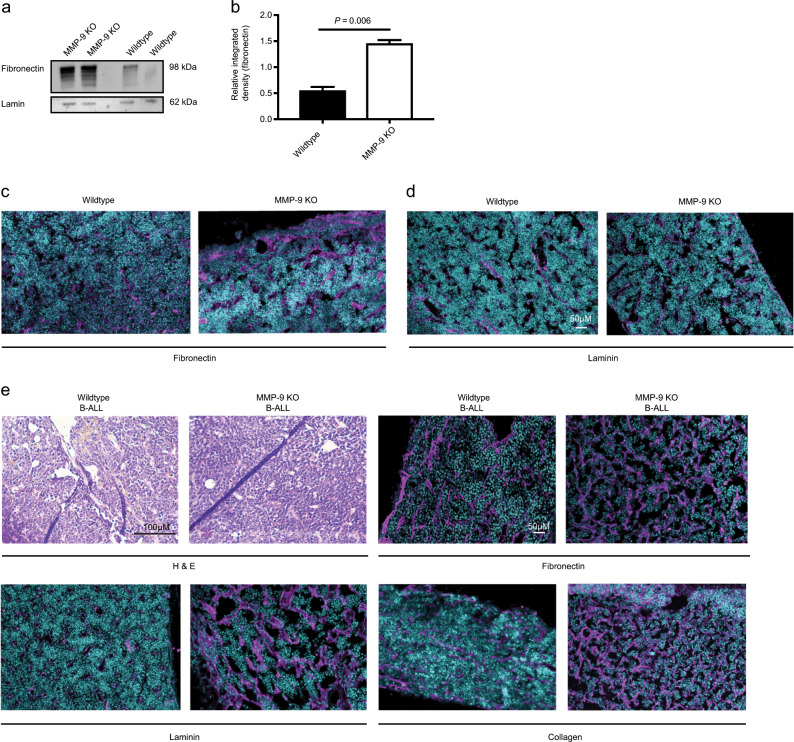
MMP-9 KO mice are characterized by an increased amount of extracellular matrix proteins in the BMM. Immunoblot (**a**) and integrated density of the highest molecular weight fibronectin band (**b**) from **a** showing fibronectin bands of varying molecular weights in protein lysates from crushed bones of wild type versus MMP-9 KO mice. The bone marrow was flushed before crushing the bones (*P* = 0.006; *t* test). The immunoblot is representative of three independent experiments. **c**, **d** Representative immunofluorescence images of bone sections of wild type or MMP-9 KO mice stained with antibodies to fibronectin (pink; **c**) or laminin (pink; **d**). The scale bar depicts 50 μM. *n* = 3–4. **e** Bone sections of wild type or MMP-9 KO mice transplanted with BCR-ABL1- transduced bone marrow in the B-ALL model stained with hematoxylin and eosin (H&E) (top left) or antibodies to fibronectin (top right), laminin (bottom left) or collagen (bottom right) in immunofluorescence studies. The scale bar depicts 100 μM in the H&E-stained slides and 50 μM in the immunofluorescence studies.

To investigate the impact of MMP-9-deficiency on levels of ECM proteins in the BMM, we performed immunoblotting of total protein lysates from crushed bones of wild type versus MMP-9-deficient mice probing with an antibody to fibronectin, a substrate of MMP-9. This (*P* = 0.006, Fig. 2a, b) and immunofluorescence staining (*P* = 0.021, Figs. 2c and S4A) revealed increased levels of fibronectin in the samples derived from MMP-9-deficient mice. Consistently, another ECM protein, laminin, (*P* = 0.02, Figs. 2d and S4B) was also increased in bone sections of MMP-9-deficient compared to wild type mice. As laminin is also expressed by endothelial basement membranes [24], we evaluated CD31^+^ endothelial cells and observed a significant reduction of CD31^+^ cells in the BM of MMP-9 KO compared with wild type mice (*P* < 0.0001, Fig. S4C–D). We confirmed increased levels of fibronectin (*P* = 0.002, Figs. 2e and S4E), laminin (*P* = 0.043, Figs. 2e and S4F) and collagen (*P* = 0.061, Figs. 2e and S4G) in MMP-9-deficient compared with wild type mice with B-ALL (Fig. 2e). These data suggest that MMP-9-deficiency in the BMM increases the levels or decreases the degradation of ECM proteins in homeostatic conditions, as well as in leukemia.

### MMP-9 promotes the invasion of B-ALL cells

**Fig. 3:**
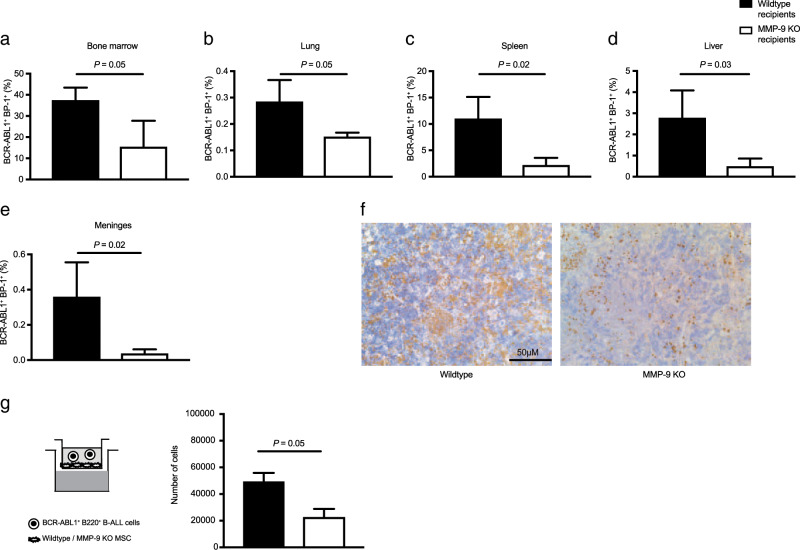
MMP-9-deficieny in recipients of B-ALL-initiating cells leads to reduced infiltration of organs. (**a**–**e**) Percentage of GFP^+^ (BCR-ABL1^+^) BP-1^+^ cells in bone marrow (**a**), lung (**b**), spleen (**c**), liver (**d**), and meninges (**e**) of wild type (black) or MMP-9 KO (white) mice transplanted with BCR-ABL1-transduced bone marrow on day 20 after transplantation (*n* = 4, *t* test, *P* values as indicated). **f** Representative immunohistochemistry images of GFP^+^ (BCR-ABL1^+^) cells (detected by immunoperoxidase using yellow–brown horseradish-peroxidase chromogen) in the meninges of wild type (left) or MMP-9 KO (right) mice transplanted with BCR-ABL1-transduced bone marrow on day 20 after transplantation. The scale bar depicts 50 μM. *n* = 4. **g** Number of primary sorted murine BCR-ABL1^+^ BP-1^+^ B-ALL cells which migrated from the upper chamber through a layer of wild type (black) or MMP-9 KO (white) mesenchymal stromal cells (MSC) to the lower chamber in a transwell/invasion assay containing 0.5 ng/ml SDF-1 (*P* = 0.05; *t* test, *n* = 2). 10^5^ leukemia cells had been plated and were allowed to migrate for 48 h.

We hypothesized that BMM-derived MMP-9 also has a role in promoting the invasion of B-ALL cells. Indeed, the preosteoblastic cell line MC3T3, MSC, macrophages from the BMM, and—to a much lesser extent—the endothelial cell line H5V expressed MMP-9 (Fig. S4H). Further, the percentage of BCR-ABL1^+^ (GFP^+^) BP-1^+^ cells in BM (*P* = 0.05, Fig. 3a) was reduced, which is likely causative of the survival prolongation in secondary recipient mice transplanted with BM from MMP-9-deficient mice with B-ALL (Fig. 1d). BCR-ABL1^+^ (GFP^+^) BP-1^+^ cells were also reduced in lung (*P* = 0.05, Fig. 3b), spleen (*P* = 0.02, Fig. 3c), liver (*P* = 0.03, Fig. 3d), and meninges (*P* = 0.02, Figs. 3e, f and S4I) of MMP-9-deficient compared with wild type mice with B-ALL.

Macrophages and neutrophils from the BMM were unlikely to be contributing to MMP-9-production [18], as they were reduced to ~5% of total BM cells in wild type mice with B-ALL (Fig. S4J). They were largely negative for GFP (BCR-ABL1) and, therefore, MMP-9 deficient in MMP-9 KO mice (Fig. S4K). In an in vitro transwell-based invasion assay we revealed reduced migration of B-ALL cells, if they had been cocultured on MMP-9-deficient compared with wild type MSC, to the chemoattractant stromal-derived factor (SDF)-1α (*P* = 0.05, Figs. 3g and S5A–B) or the B-cell chemoattractant C-X-C motif chemokine ligand 13 (CXCL13) (*P* = 0.013, Fig. S6A), for which B-ALL cells express the corresponding C-X-C motif chemokine receptor 5 (CXCR5) (Fig. S6B). In contrast, migration of B-ALL cells through a layer of CD11b^+^ F4/80^+^ wild type or MMP-9-deficient macrophages [25], known to be producers of MMP-9 [18] and potential contaminants in stromal cultures [26], did not differ significantly (Fig. S6C–D). In summary, BMM-derived MMP-9 promotes the migration or invasion of B-ALL cells into different peripheral organs, possibly via degradation of the ECM.

### B-ALL cells instruct the BMM to produce MMP-9 via the release of tumor necrosis factor α

**Fig. 4:**
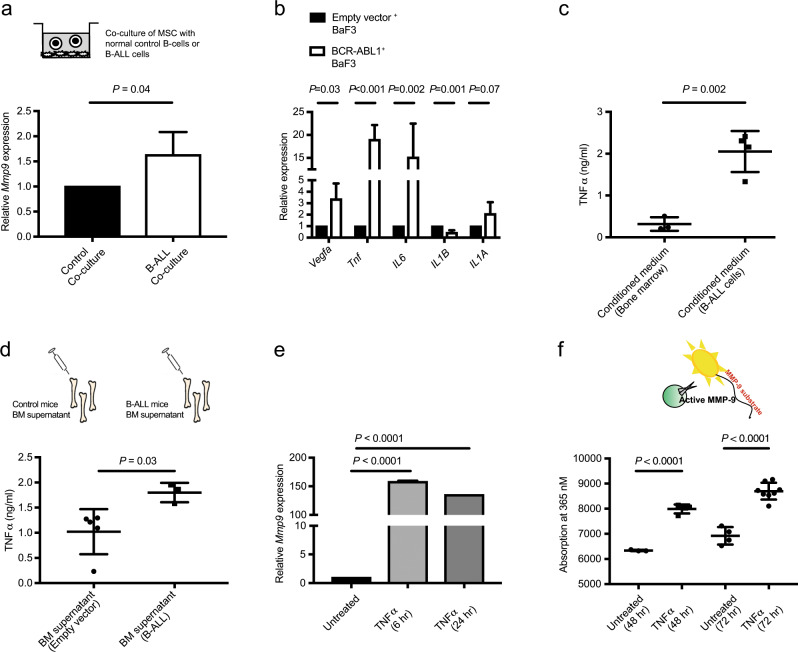
B-ALL-derived *Tnf*α remodels the bone marrow microenvironment. **a** Relative expression of *Mmp9* in wild type mesenchymal stromal cells after 48 h of coculture with 10^5^ normal B cells (black), which had been enriched by anti-BP-1 magnetically labeled antibodies, or whole bone marrow from mice with fully established B-ALL (>95% BP-1^+^ GFP^+^ (BCR-ABL1^+^) cells in the bone marrow) (white). (*P* = 0.04; *t* test, *n* = 4–6). **b** Relative expression of *Vegfa, Tnf, IL6, IL1B,* and *IL1A* in BaF3 cells transduced with empty vector (black)- or BCR-ABL1 (white)-expressing retrovirus (*P* values as indicated; *t* test, *n* = 3–5). **c** Concentration of *Tnf*α in ng/ml in the CM from cultured bone marrow cells from nonirradiated normal mice (black circles) or mice with B-ALL (black squares). The CM was harvested after 7 days of culture, and *Tnf*α was detected by ELISA (*P* = 0.002; *t* test, *n* = 4). **d** Concentration of *Tnf*α in the bone marrow supernatant, harvested by flushing bones with 100 μl of PBS from wild type mice transplanted with empty vector (black circles)- or BCR-ABL1 (black squares)-transduced bone marrow (*P* = 0.03; *t* test, *n* = 4–5). *Tnf*α was detected by ELISA. **e** Relative expression of *Mmp9* in MSC after no treatment (black) or after in vitro treatment with 15 ng/ml *Tnf*α for 6 (lighter gray) or 24 h (darker gray) (*P* < 0.0001; ANOVA, Tukey Test, *n* = 4–5). **f** Absorbance (at 365 nm) of fluorescent, emitted light after cleavage of an MMP-9-specific fluorogenic substrate, which had been incubated with the conditioned medium from MSC, that were untreated or treated with 15 ng/ml *Tnf*α (*P* < 0.0001; *t* test, *n* = 4–5). The conditioned medium had been harvested 48 h after initiating *Tnf*α treatment, and the substrate had been incubated with the conditioned medium for 40–60 min.

We hypothesized that B-ALL cells induce the expression of *Mmp9* in the BMM. When we cocultured wild type MSC with normal BP-1-enriched B cells versus B-ALL cells, we observed increased expression of *Mmp9* by MSC after coculture with B-ALL, but not normal B cells (*P* = 0.04, Fig. 4a). A focused gene expression analysis of B-ALL cells revealed an ~15-fold increased expression of the proinflammatory cytokine *Tnf*α in BaF3 cells transduced with BCR-ABL1 compared with empty vector (*P* < 0.001, Fig. 4b). More myeloid-associated cytokines were decreased in the BM of mice with B-ALL (Fig. S6E). Consistently, protein levels of *Tnf*α in the conditioned medium (CM) harvested from cultured, primary BCR-ABL1^+^ B-ALL cells was significantly higher than in CM from healthy BM cells (*P* = 0.002, Fig. 4c). *Tnf*α levels were also higher in the BM supernatant of mice which had received BM transduced with BCR-ABL1 compared to empty vector (*P* = 0.03, Fig. 4d). Next, we investigated if increased secretion of *Tnf*α by BCR-ABL1^+^ cells may be responsible for inducing the expression of *Mmp9* in the BMM. Indeed, in vitro treatment of MSC and MC3T3 cells with recombinant *Tnf*α led to increased expression of *Mmp9* in MSC (*P* < 0.0001, Fig. 4e) and MC3T3 (*P* = 0.002, Fig. S6F). To test if increased expression of *Mmp9* in MSC after *Tnf*α treatment leads to secretion of active, functional MMP-9 protein, we performed a fluorogenic in vitro assay, in which MMP-9-dependent hydrolysis of a fluorochrome-coupled MMP-9-specific substrate liberates a fluorescent cleavage product. Hereby, we observed increased MMP-9 activity in the CM harvested from MSC (*P* < 0.001, Fig. 4f) or MC3T3 (*P* = 0.04, Fig. S6G) cells after treatment with *Tnf*α. In order to test the specific proteolytic activity of MMP-9 and MMP-2, which is predominantly produced by mesenchymal cells [27], we performed gelatin zymographies. This revealed an increase of MMP-9 activity in the CM from MSC, but not macrophages, after exposure to *Tnf*α (Fig. S7A), regardless of whether the MSC had been depleted of macrophages (Fig. S7B–C). Similar findings were observed using lysates from MSC (Fig. S7D). An increase of MMP-9 activity was also found in H5V and MC3T3 cells after treatment with *Tnf*α (Fig. S7E). MMP-2 levels remained unaltered (Figs. S7A and C–E). In an effort to exclude the possibility that macrophages or neutrophils may be contributing to MMP-9 production, we demonstrated that lysates of whole BM fluids from wild type and, more prominently, MMP-9 KO mice transplanted with BCR-ABL1-transduced BM had lower MMP-9 activity than mice transplanted with empty vector control^+^ BM (Fig. S7F). Consistently, only the exposure of MSC, but not macrophages or neutrophils, to *Tnf*α led to increased MMP-9 activity, as performed in serum-free conditions to abolish the contribution of MMP-9 or MMP-2 in serum (Fig. S7G). In addition, the CM and lysates of normal B220^+^ B cells revealed more MMP-9 activity than BCR-ABL1^+^ B220^+^ B cells (Fig. S7H). These data suggest that B-ALL cells produce *Tnf*α (and low amounts of MMP-9), which contributes to remodeling of the BMM leading to the release of functional MMP-9, predominantly by MSC.

### B-ALL cell-derived *Tnf*α activates the *Tnf* receptor 1-dependent NF-κB pathway inducing Mmp9 expression in BM niche cells

**Fig. 5:**
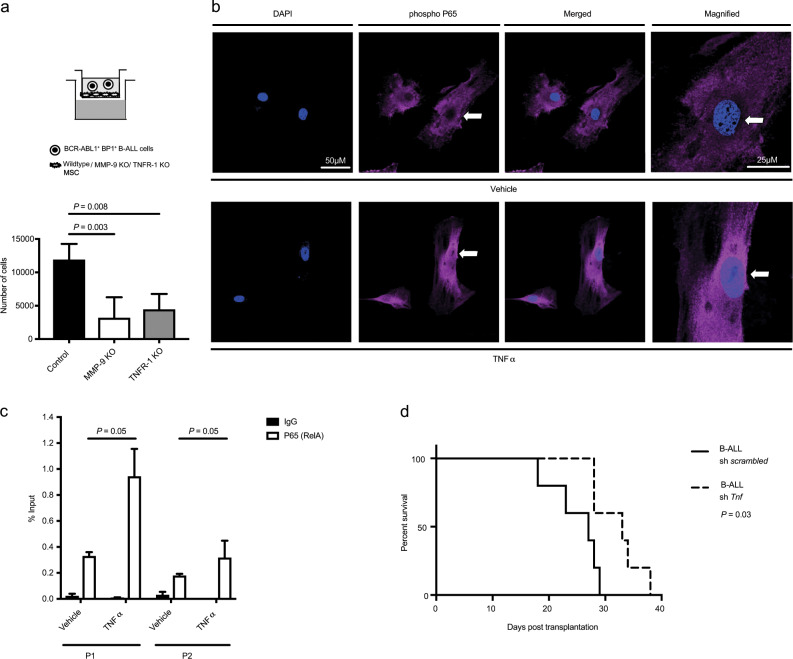
Deficiency of *Tnf*α in B-ALL cells prolongs survival. **a** Number of primary murine BCR-ABL1^+^ BP-1^+^ B-ALL cells which migrated from the upper chamber through a layer of wild type (black), MMP-9 KO (white), or tumor necrosis factor receptor (TNFR)-1 KO (gray) MSC to the lower chamber in a transwell–invasion assay, which contained 500 ng/ml CXCL13 (*P* value as indicated; ANOVA, Tukey test, *n* = 4–6). 10^5^ leukemia cells had been plated and were allowed to migrate for 24 h. **b** Representative immunofluorescence images of MSC after 8 h of treatment with vehicle (top) or *Tnf*α (bottom), stained with an antibody to phospho P65 (red) and DAPI (blue). The scale bar depicts 50 μm in all images and 25 μm in the magnified images (extreme right panel). *n* = 9–10. The images are representative of two independent experiments. **c** Binding of P65 to the *Mmp9* promoter in MSC treated with vehicle or 15 ng/ml *Tnf*α for 6 h, as measured by a ChIP assay using an anti-P65 (white) or a control IgG (black) antibody and two different primer pairs (P1 and P2) for *Mmp9* (*P* = 0.05, *t* test, *n* = 3). **d** Kaplan–Meier-style survival curve for wild type C57/Bl6 recipients of BCR-ABL1-transduced bone marrow cotransduced with *scrambled* shRNA- (solid line) or *Tnf*α shRNA-expressing lentivirus (dashed line) in the B-ALL model (*P* = 0.03, Log-rank test, *n* = 5).

*Tnf*α binds to its receptors, *Tnf* receptor (TNFR) 1 or TNFR 2, expressed on MSC. As TNFR1 has, generally, been implicated in proinflammatory conditions [28], we isolated MSC from TNFR1-deficient mice and treated them with *Tnf*α in vitro. This did not lead to increased expression of *Mmp9* in TNFR1 deficient MSC (Fig. S8A) compared with untreated controls or compared with wild type MSC treated with *Tnf*α (Fig. 4e). The presence of TNFR1 deficient MSC in the transwell–invasion assay also led to a reduction of the invasive ability of B-ALL cells (*P* = 0.008, Fig. 5a), suggesting that *Tnf*α signals to MSC via TNFR1. We stained MSC for components of the nuclear factor kappa-light-chain-enhancer of activated B cells (NF-κB) protein complex, which had previously been implicated in *Tnf*α-mediated signaling [29, 30], to test its possible role for inducing *Mmp9* expression. Indeed, we observed increased nuclear translocation and staining for phospho P65 (RelA), a transcription factor and activating partner in the NF-κB complex, in MSC treated with *Tnf*α compared with vehicle (*P* = 0.017, Figs. 5b and S8B). To test, if NF-κB was directly inducing the expression of *Mmp9* in MSC via binding to the MMP-9 promoter, we performed chromatin immunoprecipitation (CHIP) with an antibody to P65 on lysates from MSC treated with vehicle or *Tnf*α. This revealed that treatment with *Tnf*α significantly increased the binding of P65 to the *Mmp9* promoter (*P* = 0.05, Fig. 5c), but binding to a control region (located on chromosome 18) was undetectable.

Transplantation of BM cotransduced with BCR-ABL1-expressing retrovirus and *scrambled*- versus *Tnf*-shRNA-expressing lentivirus led to a significant prolongation of survival of wild type recipient mice transplanted with *Tnf*α shRNA- compared with *scrambled* shRNA-expressing BCR-ABL1^+^ LIC (*P* = 0.03, Figs. 5d and S8C). Consistently, culturing of MSC, macrophages, or neutrophils in the CM of BCR-ABL1^+^ BaF3 cells transduced with *Tnf*α shRNA-expressing lentivirus (Fig. S8D) led to a reduction of MMP-9 levels compared with cells cultured in the CM from *scrambled* shRNA^+^ BCR-ABL1^+^ BaF3 cells (Fig. S8E). Taken together our data suggest that *Tnf*α, derived from B-ALL cells and contributing to B-ALL progression, binds to its receptor TNFR1 activating the NF-κB protein complex. P65 as a component of the activated NF-κB complex binds to the promoter of *Mmp9*, inducing its expression.

### Pharmacological inhibition of MMP-9 significantly prolongs survival of mice with B-ALL

**Fig. 6:**
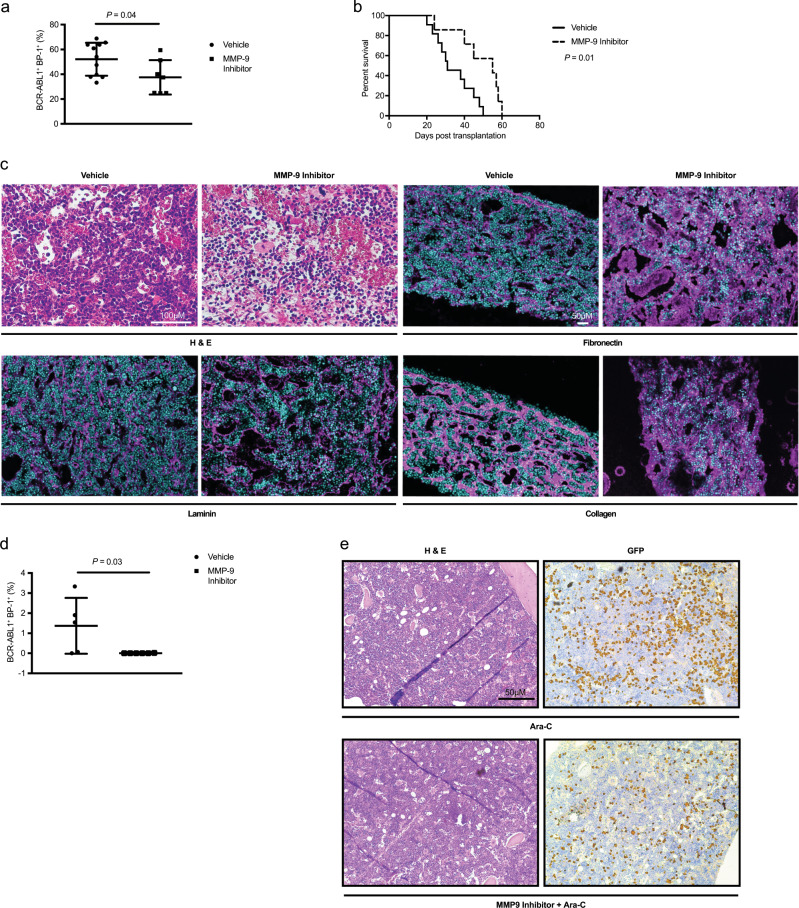
Treatment with an MMP-9-inhibitor prolongs survival in B-ALL. **a** Percentage of GFP^+^ (BCR-ABL1^+^) BP-1^+^ cells in peripheral blood of vehicle (black circles)- or MMP-9 inhibitor (black squares)-treated mice on day 25 of transplantation of BCR-ABL1-transduced bone marrow (*P* = 0.04; *t* test, *n* = 7–11). **b** Kaplan–Meier-style survival curve of vehicle (solid line)- or MMP-9 inhibitor (dashed line)-treated mice transplanted with 1 × 10^6^ BCR-ABL1-transduced bone marrow cells (*P* = 0.01, Log-rank test, *n* = 7–11). The mice were treated with the MMP-9 inhibitor 2–3 times per week at a dose of 20 mg/kg beginning at day 12 after transplantation. **c** Bone sections of mice with B-ALL treated with vehicle or the MMP-9 inhibitor stained with hematoxylin and eosin (H&E) (top left) or antibodies to fibronectin (pink; top right), laminin (pink; bottom left), or collagen (pink; bottom right) in immunofluorescence studies. The dosing of the MMP-9 inhibitor was as described in **b**. The scale bar depicts 100 μm in the slides stained with H&E and 50 μm in the immunofluorescence studies. **d** Percentage of GFP^+^ (BCR-ABL1^+^) BP-1^+^ lymphoid cells in peripheral blood of wild type, untreated secondary recipients of BCR-ABL1^+^ bone marrow from vehicle (black circles)- or MMP-9 inhibitor (black squares)-treated primary mice with B-ALL on day 18 after transplantation (*P* = 0.03; *t* test, *n* = 5). **e** Representative images of bone sections of mice with B-ALL treated with the chemotherapeutic agent cytarabine (top) (ara-C; 50 mg/kg from day 12 for 5 days, followed by 2 weeks rest) or the MMP-9 inhibitor (20 mg/kg) and cytarabine (bottom) stained with hematoxylin and eosin (H&E) (left) or anti-GFP (detected by immunoperoxidase using yellow–brown horseradish-peroxidase chromogen; BCR-ABL1^+^ B-ALL cells; right) by immunohistochemistry. The scale bar depicts 50 μm.

Next, we evaluated if inhibition of MMP-9 may be exploited therapeutically for the treatment of B-ALL. Inhibition of MMP-9 with a pharmacological inhibitor significantly reduced the percentage of BCR-ABL1^+^ (GFP^+^) BP-1^+^ cells in peripheral blood compared with vehicle-treated mice (*P* = 0.04, Fig. 6a), and significantly prolonged survival (*P* = 0.01, Fig. 6b). To confirm that the survival prolongation of mice with B-ALL treated with an MMP-9 inhibitor was indeed due to reduced degradation of the ECM in the BMM, we performed immunofluorescence staining of bone sections. We observed reduced leukemic infiltration (Fig. 6c) and significantly increased levels of fibronectin (*P* = 0.001, Figs. 6c and S9A), collagen (*P* = 0.05, Figs. 6c and S9B), and laminin (*P* *=* 0.002, Figs. 6c and S9C) in the BMM of mice with B-ALL after treatment with an MMP-9 inhibitor compared with vehicle. We performed a secondary transplantation and observed a significant reduction of leukemia load in the peripheral blood of secondary recipient mice transplanted with BM from MMP-9 inhibitor-treated primary B-ALL mice compared with controls (*P* = 0.03, Fig. 6d). However, disease induction in secondary recipients was inefficient, as can be observed in this B-ALL model (DSK, unpublished data), and no mice succumbed to B-ALL. In vitro plating of B-ALL cells from vehicle- versus MMP-9-inhibitor-treated mice on wild type MSC in limiting dilution to test proliferation of B-ALL cells demonstrated reduced cell proliferation if the cells were derived from an MMP-9 inhibitor-derived BMM (*P* = 0.009, Fig. S9D). Hypothesizing that MMP-9 inhibition in conjunction with standard chemotherapy with cytarabine (ara-C) may reduce the tumor load more efficiently than ara-C alone, we revealed a trend toward a reduced tumor burden by the MMP-9 inhibitor alone and a significant reduction of the tumor burden by ara-C alone or ara-C in combination with the MMP-9 inhibitor in peripheral blood (Fig. S10A), BM (Fig. S10B), and spleen (Fig. S10C), but not in lung (Fig. S10D). Spleen weights were significantly reduced in mice treated with ara-C or ara-C and the MMP-9 inhibitor (Fig. S10E). In all examined tissues the combination treatment was not more effective than ara-C alone, which alone was so potent, that it effectively eradicated the disease and prevented death due to leukemia. However, testing minimal residual disease (MRD), which is responsible for disease relapse in patients with B-ALL, at the time of death a reduction of GFP^+^ (BCR-ABL1^+^) MRD was found in the BM when mice with B-ALL had been treated with ara-C and the MMP-9 inhibitor compared with mice treated with ara-C alone (*P* = 0.003, Figs. 6e and S10F). Disease burden was similar between treatment groups prior to the start of therapy (Fig. S10G). In summary, treatment of B-ALL mice with an MMP-9 inhibitor prolonged survival of mice with B-ALL, likely via reduction of the degradation of ECM proteins, without overt toxicity. However, at the chosen dose of ara-C, the tumor burden could not be further reduced or survival prolonged when ara-C was combined with an MMP-9-inhibitor. Our data suggest, that inhibition of MMP-9 may be a feasible adjunct to existing therapies of B-ALL, while also reducing MRD.

### Levels of fibronectin in bone sections of patients with B-ALL are reduced

**Fig. 7:**
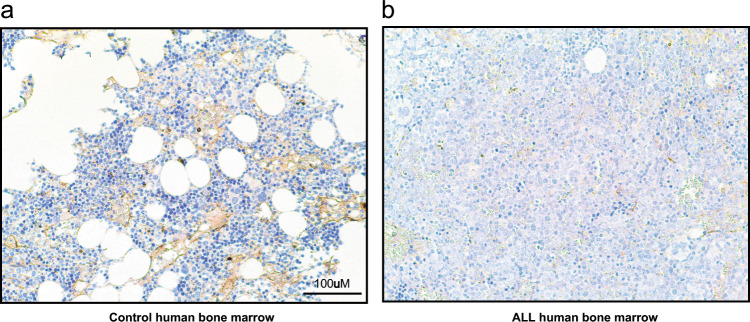
Fibronectin levels may be reduced in bone sections of patients with B-ALL. Representative images of bone sections of a healthy individual (control; left) versus a patient with B-ALL (right) stained with an antibody to fibronectin (detected by immunoperoxidase using yellow–brown horseradish-peroxidase chromogen) in immunohistochemistry studies. The scale bar depicts 100 μm.

In human samples we revealed a decreased amount of fibronectin in bone sections of patients with B-ALL compared with healthy controls (Fig. 7a). Levels of fibronectin in bone sections of patients with B-ALL were also significantly reduced compared with bone sections of patients with CML (Fig. S11A). In contrast, the concentration of MMP-9 in the plasma of BM aspirates was highest in patients with CML versus healthy controls and patients with B-ALL (Fig. S11B). As revealed by the punctate staining pattern for MMP-9 by immunohistochemistry, however, these high levels of MMP-9 in healthy controls and CML patients were likely due to MMP-9 production by myeloid cells compared with a more diffuse staining pattern in B-ALL patients (Fig. S11C). These data suggest that the concept of decreased ECM proteins in a B-ALL BMM, possibly leading to increased invasion of B-ALL cells and leukemia progression, may also apply to the clinical setting. However, in healthy controls and CML patients MMP-9 is largely produced by myeloid cells.

## Discussion

Here we report that BCR-ABL1^+^ B-ALL cells remodel the BM niche via the release of *Tnf*α, which activates the NF-kB pathway downstream of TNFR1 leading to increased expression and release of MMP-9. Increased production of functional MMP-9 in the BMM degrades ECM proteins such as fibronectin, laminin, and collagen facilitating the migration/invasion of B-ALL cells in the BM, as well as in different organs in our murine model. Reduced infiltration of the BM and peripheral organs by leukemic cells decreases disease aggressiveness and, consequently, prolongs the survival of mice with B-ALL. Our results also demonstrate that inhibition of MMP-9 can be used therapeutically in our murine model of B-ALL without overt toxicity.

Most previous reports on the role of MMP-9 in cancer have focused on the cancer cell-specific production and release of MMP-9 leading to increased invasion [16, 31] and progression [32], both in solid tumors [33], as well as in leukemia [23, 34]. MMP-9 production in solid tumors has also been associated with increased metastasis [35], and in B-cell CLL levels of MMP-9 in plasma correlate with prognosis [36]. In a large proportion of B-ALL cases, leukemia cell-specific production of MMP-9 has been shown [37], as also described in the BloodSPot Database and found to a low extent in our study. However, as demonstrated in our data, leukemia cell-specific production of MMP-9 does not influence survival (Fig. S1C), suggesting that the *Tnf*α-induced production of MMP-9 by MSC and possibly other niche cells plays a more prominent role. Production and release of pro-MMP-9, which is activated to functional MMP-9, has been shown to lead to degradation of ECM proteins enhancing cancer progression [38], similar to our study. However, the source of MMP-9 may also lie in the BMM, as our data show. Further, our findings are consistent with several other studies demonstrating the ability of leukemia cells to remodel the BMM into a leukemia-promoting and -perpetuating niche via the release of cytokines [2, 3, 39], extracellular vesicles [40] or other pathways [41]. Whether our observations are specific to BCR-ABL1^+^ leukemia will be assessed in future.

Several chronic inflammatory conditions such as inflammatory bowel disease or chronic infection by helicobacter pylori and others have been associated with cancer [42]. A role of *Tnf*α for these inflammation-associated cancers has been implicated, as mice deficient for *Tnf*α have reduced susceptibility for skin cancers [43]. In pancreatic cancer *Tnf*α as a proinflammatory cytokine has been strongly associated with increased invasion, and inhibition of *Tnf*α led to antitumoral effects [44], while blockade of *Tnf*α reduced carcinogenesis in a model of chronic colitis [45]. In agreement with our study autocrine secretion of *Tnf*α has been shown to support and maintain leukemia cells in CML [46], and *Tnf*α treatment of leukemia cells is known to lead to NFκB-mediated transcription of MMP-9 [47]. However, the effect of *Tnf*α on remodeling of the BMM had not been investigated in the setting of CML.

In agreement with the studies by Schepers et al. in CML [48] or by Arranz et al. in myeloproliferative neoplasia [39], we demonstrate that leukemia cells produce inflammatory cytokines, with *Tnf*α being one of the main mediators of niche remodeling in B-ALL. However, the involvement of other inflammatory cytokines released from B-ALL cells for the remodeling of the BMM cannot be excluded and needs to be addressed in future. While the knockdown of *Tnf*α in leukemia cells in our study prolonged the survival of mice with B-ALL by a concomitant reduction in MMP-9 production by the BMM (Fig. S8E), the impairment of autocrine, leukemia-promoting secretion of *Tnf*α, as shown in CML [46], may be contributory.

Relapse after first line therapies is a major drawback in various cancer treatments, which holds true in the case of B-ALL. MRD, including residual leukemia cells in the central nervous system [49], is known to be associated with relapse in B-ALL patients [50]. While toxic effects from inhibition of MMP-9 led to the failure of this treatment in various solid tumor therapies, we observed that B-ALL cells—possibly in conjunction with cytarabine—are highly sensitive to MMP-9 inhibition at lower doses, which led to reduced leukemia progression but no overt toxicity. MMP-9 inhibition also efficiently reduced MRD, including in the “sanctuary” of the central nervous system, i.e., the meninges. Therefore, careful exploration of the clinical benefit of MMP-9 inhibition at low dose or a therapeutic decrease of MMP-9 levels by other methods such as by RNA silencing therapeutics for reduction of MRD in B-ALL may be a worthwhile cause. Hereby, inhibition of cancer cell-intrinsic production and secretion of MMP-9, of course, cannot be ruled out.

The higher levels of fibronectin in the BMM and higher MMP-9 levels in the plasma of BM aspirates of healthy individuals and CML compared with B-ALL patients may seem contradictory, but may be explained by the increased production of MMP-9 by myeloid cells [18], the production of fibronectin by CML cells [51], or by other compensatory factors.

In summary, our data suggest that a proinflammatory state is created in the leukemic BMM in B-ALL by the release of *Tnf*α, which leads to remodeling of the BMM, breakdown of the ECM, and increased invasion of leukemia cells via the secretion of MMP-9 from MSC. Therapeutic administration of an MMP-9 inhibitor at lower dose in conjunction with chemotherapeutic agents may counteract this pathway, decrease MRD and prolong survival in patients.

## Supplementary information

Supplementary figures, tables and methods
