# Supplementary material for: Bone marrow niche-derived extracellular matrix-degrading enzymes influence the progression of B-cell acute lymphoblastic leukemia
Source: Leukemia. 2020 Jan 9;34(6):1540–52. doi: 10.1038/s41375-019-0674-7 (PMC7266746; doi:10.1038/s41375-019-0674-7)
Supplement: Supplementary file 1 — Supplementary figures, tables and methods [file 41375_2019_674_MOESM1_ESM.pdf]

Supplementary figure 1

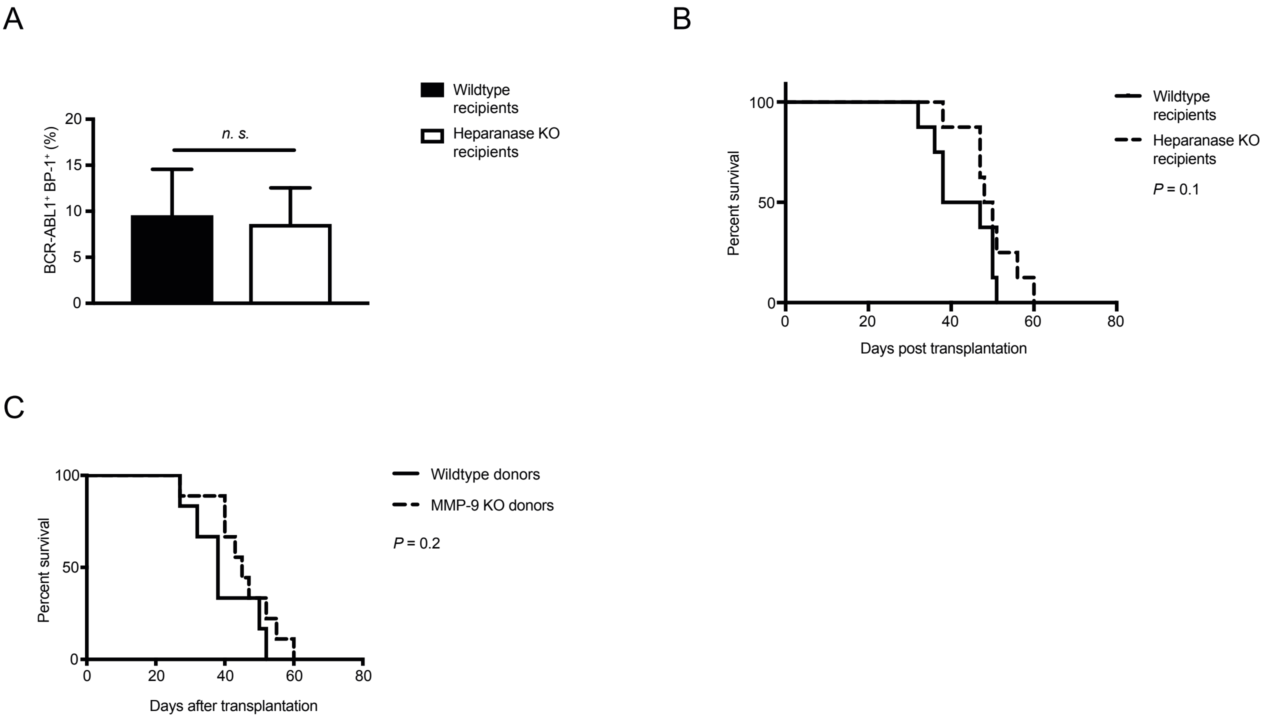

Supplementary Figure 1: A) Percentage of GFP⁺ (BCR-ABL1⁺) BP-1⁺ lymphoid cells in peripheral blood of wildtype (black) or heparanase KO (white) recipient mice on day 25 after transplantation of BCR-ABL1-transduced bone marrow (t-test, n=8). B) Kaplan-Meier-style survival curve of wildtype (solid line) or heparanase KO (dashed line) recipient mice transplanted with 1 x 10^6 BCR-ABL1-transduced bone marrow cells in the B-ALL model (Log-rank test, n=8). C) Kaplan-Meier-style survival curve of wildtype recipient mice transplanted with 1 x 10^6 BCR-ABL1-transduced wildtype (solid line) or MMP-9 KO (dashed line) bone marrow in the B-ALL model (Log-rank test, n=8).

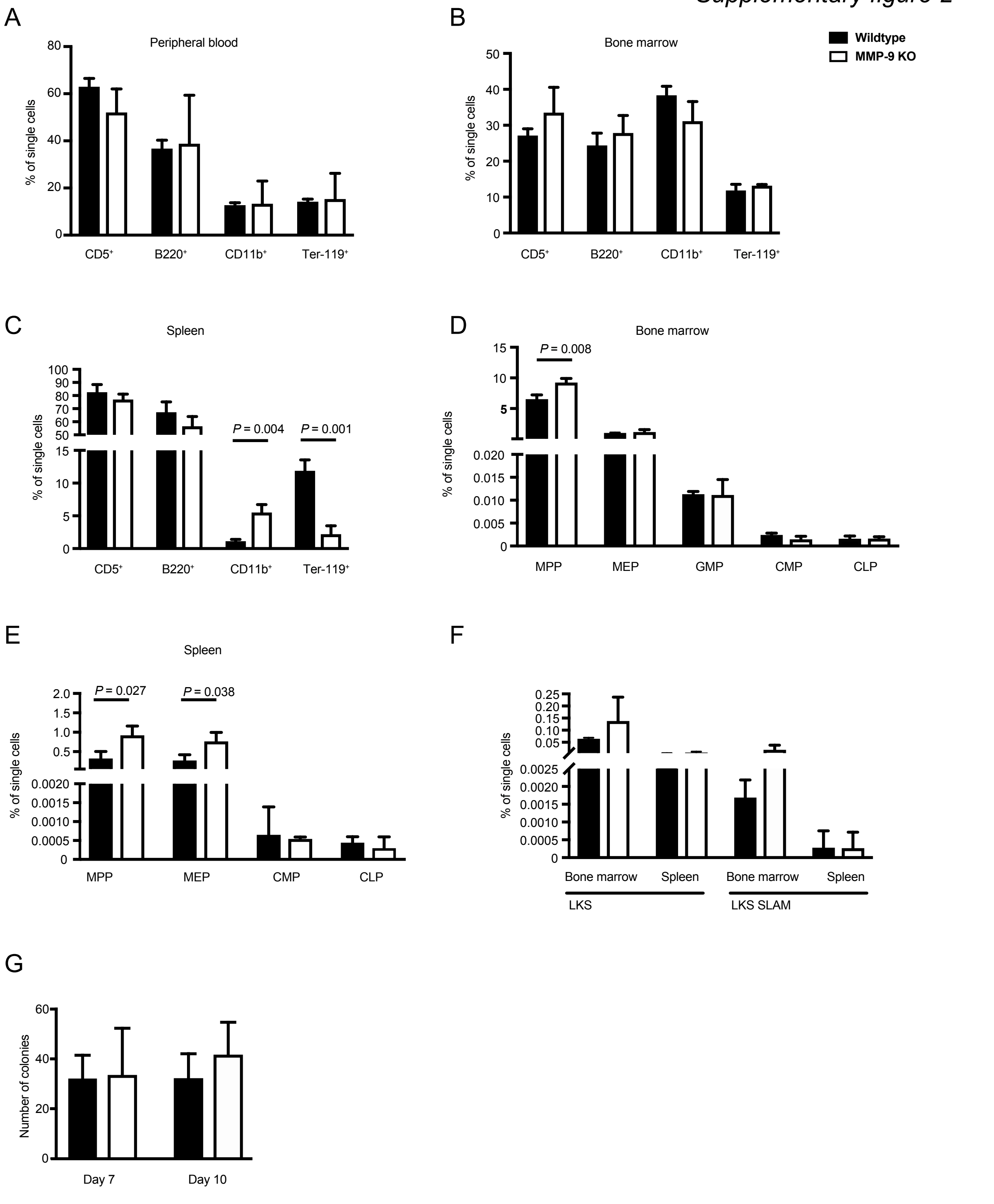

Supplementary Figure 2: A-C) Percentage of CD5⁺, B220⁺, CD11b⁺ or Ter-119⁺ cells in peripheral blood (A), bone marrow (B) or spleen (C) of healthy wildtype (black) or MMP-9 KO mice (white) (n=3, t-test). D-E) Percentage of MPP, MEP, GMP, CMP and CLP in bone marrow (D) and spleen (E) of healthy wildtype (black) or MMP-9 KO mice (white) (n=3, t-test). No GMP were detected in spleen. F) Percentage of LKS and LKS SLAM cells in bone marrow and spleen of healthy wildtype (black) and MMP-9 KO mice (white) (n=3, t-test). G) Number of colonies from total bone marrow of healthy wildtype (black) or MMP-9 KO mice (white) in methylcellulose on days 7 and 10 after plating. 10 × 10^4 total bone marrow cells had been plated. Each murine bone marrow sample (n=3, t-test) had been plated in triplicate.

Supplementary figure 3

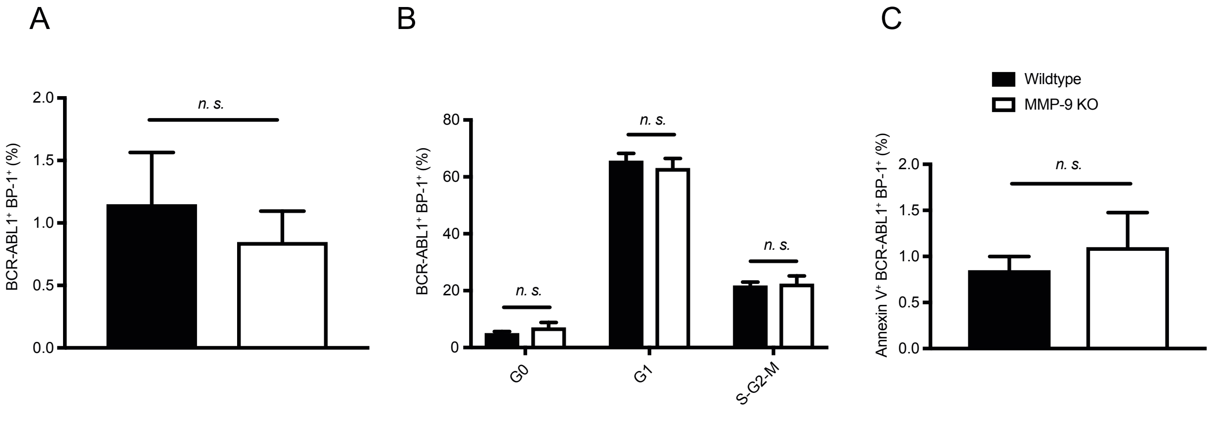

Supplementary Figure 3: A) Percentage of GFP+ (BCR-ABL1+) BP-1+ cells of total leukocytes which homed to the bone marrow of wildtype (black) or MMP-9 KO (white) recipient mice 18 hours after transplantation (t-test, n=5). B-C) Percentage of GFP+ (BCR-ABL1+) BP-1+ cells in the G0, G1, S-G2-M phase of the cell cycle (One-way ANOVA, Tukey Test) (B) and percentage of GFP+ (BCR-ABL1+) BP-1+ annexin V+ DAPI+ cells (C) in the bone marrow of wildtype (black) or MMP-9 KO (white) mice with B-ALL on day 20 after transplantation (t-test, n=3-4).

Supplementary figure 4

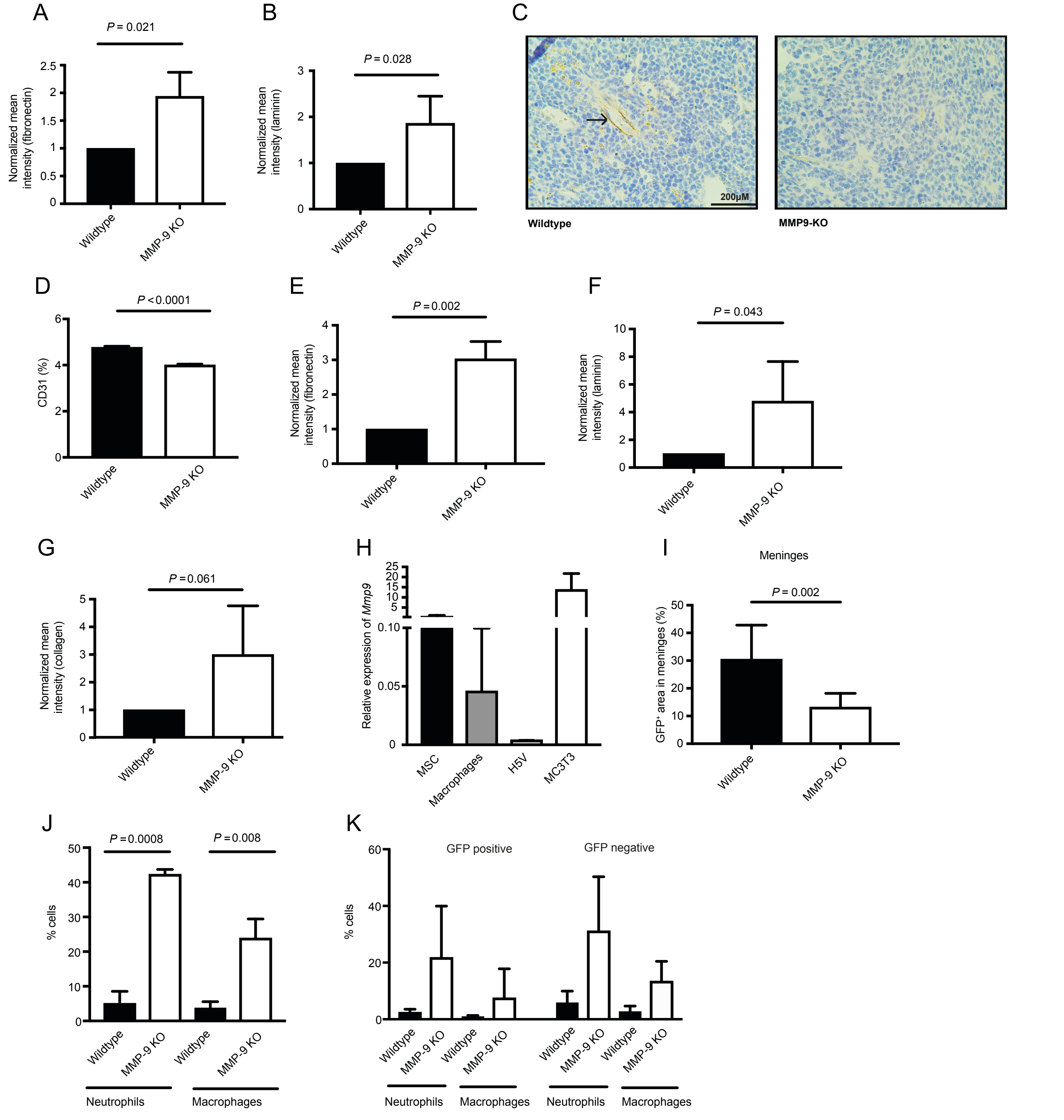

Supplementary Figure 4: A-B) Normalized mean intensity of the extracellular matrix proteins fibronectin (*P* = 0.021; t-test) (A) or laminin (*P* = 0.028; t-test) (B) in bone sections of healthy wildtype (black) or MMP-9 KO (white) mice (n=3-4). The quantifications were performed with Fiji. C) Representative immunohistochemistry images of bone sections from wildtype or MMP-9 KO mice with B-ALL stained with an antibody to CD31 (detected by immunoperoxidase using yellow-brown horseradish-peroxidase chromogen). The arrow indicates CD31$^+$ endothelial cells. The scale bar depicts 100µm. D) Percentage of CD31$^+$ cells in bone marrow of healthy wildtype (black) or MMP-9 KO mice (white) by flow cytometry (*P* < 0.0001; t-test, n=3). (E-G) Normalized mean intensity of the extracellular matrix proteins fibronectin (*P* = 0.002; t-test) (E), laminin (*P* = 0.043; t-test) (F) or collagen (*P* = 0.061; t-test) (G) in bone sections of wildtype or MMP-9 KO mice with B-ALL on day 20 after transplantation (n=3-4). H) Relative expression of *Mmp9* in wildtype MSC (black), macrophages (dark gray), H5V (light gray) and MC3T3 cells (white). I) Percentage of the GFP$^+$ area of the entire area in meninges of wildtype (black) or MMP-9 KO (white) recipient mice transplanted with BCR-ABL1$^+$ bone marrow cells on day 20 after transplantation (*P* = 0.002, t-test, n=4). The quantification was performed using the Fiji imaging tool on representative immunohistochemistry images of the meninges. J-K) Percentage of total Gr-1$^+$ neutrophils and CD11b$^+$ F4/80$^+$ macrophages (J) and percentage of neutrophils or macrophages as in (J), divided by positivity for GFP (BCR-ABL1), (K) in total bone marrow of wildtype (black) or MMP-9 KO (white) recipient mice transplanted with BCR-ABL1-transduced bone marrow on day 22 after transplantation (One-way ANOVA; Tukey Test; n=3).

Supplementary figure 5

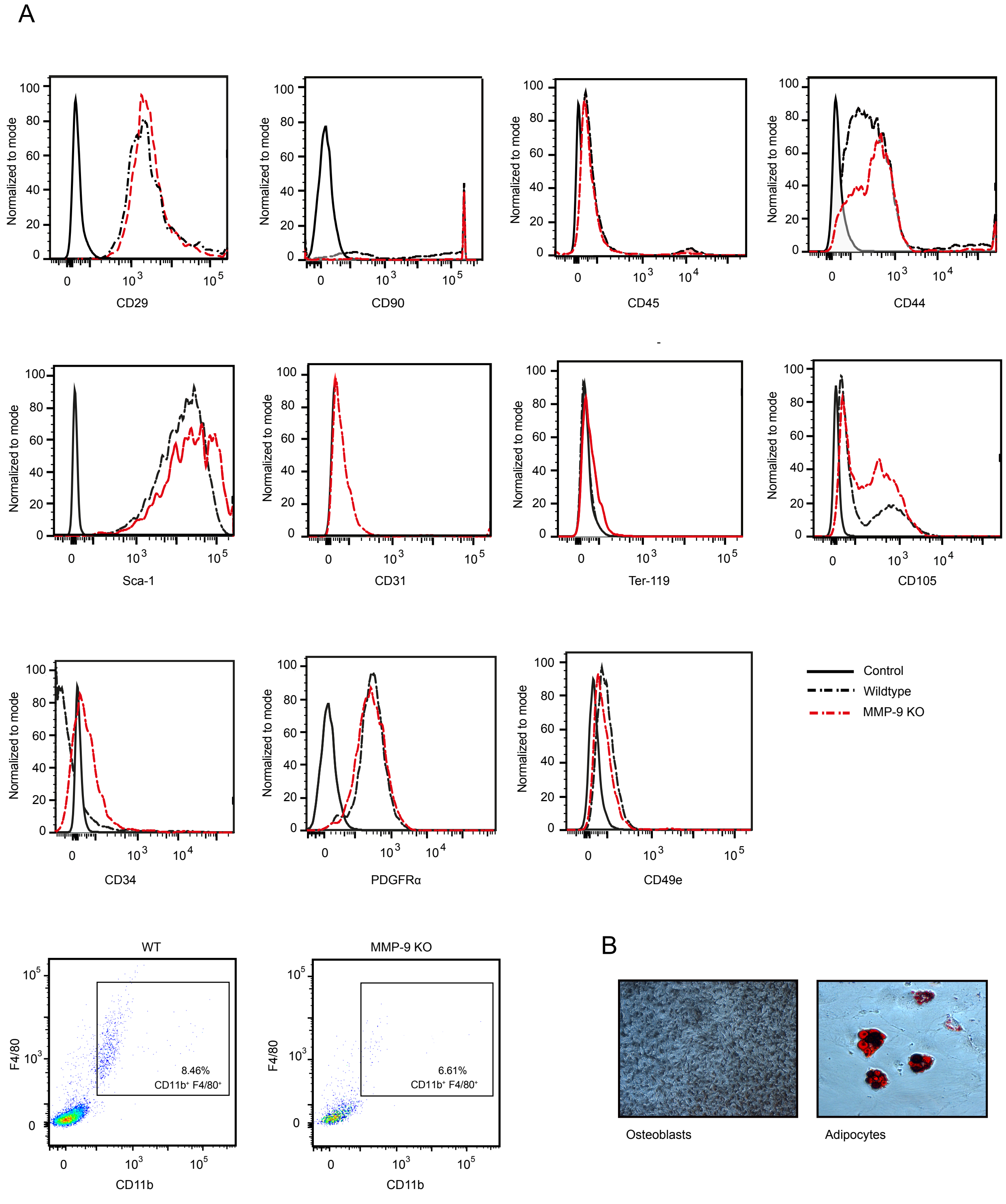

Supplementary figure 5: A) Representative images of the expression of CD29, CD90, CD45, CD44, Sca-1, CD31, Ter-119, CD105, CD34, PDGFR α, CD49e and CD11b$^+$ F4/80$^+$ on wildtype (black dashed line) versus MMP-9 KO (red dashed line) MSC at passage 4-5 versus unstained control (solid black line). The percentages of CD11b$^+$ F4/80$^+$ in wildtype and MMP-9 KO are 8.46% and 6.61%, respectively. The expression has been normalized to mode. B) Differentiation of sorted wildtype MSC to osteoblasts (left) and adipocytes (right). Osteoblasts are stained with Von Kossa, and adipocytes are stained with Oil Red O. The images are representative of 3 independent experiments.

Supplementary figure 6

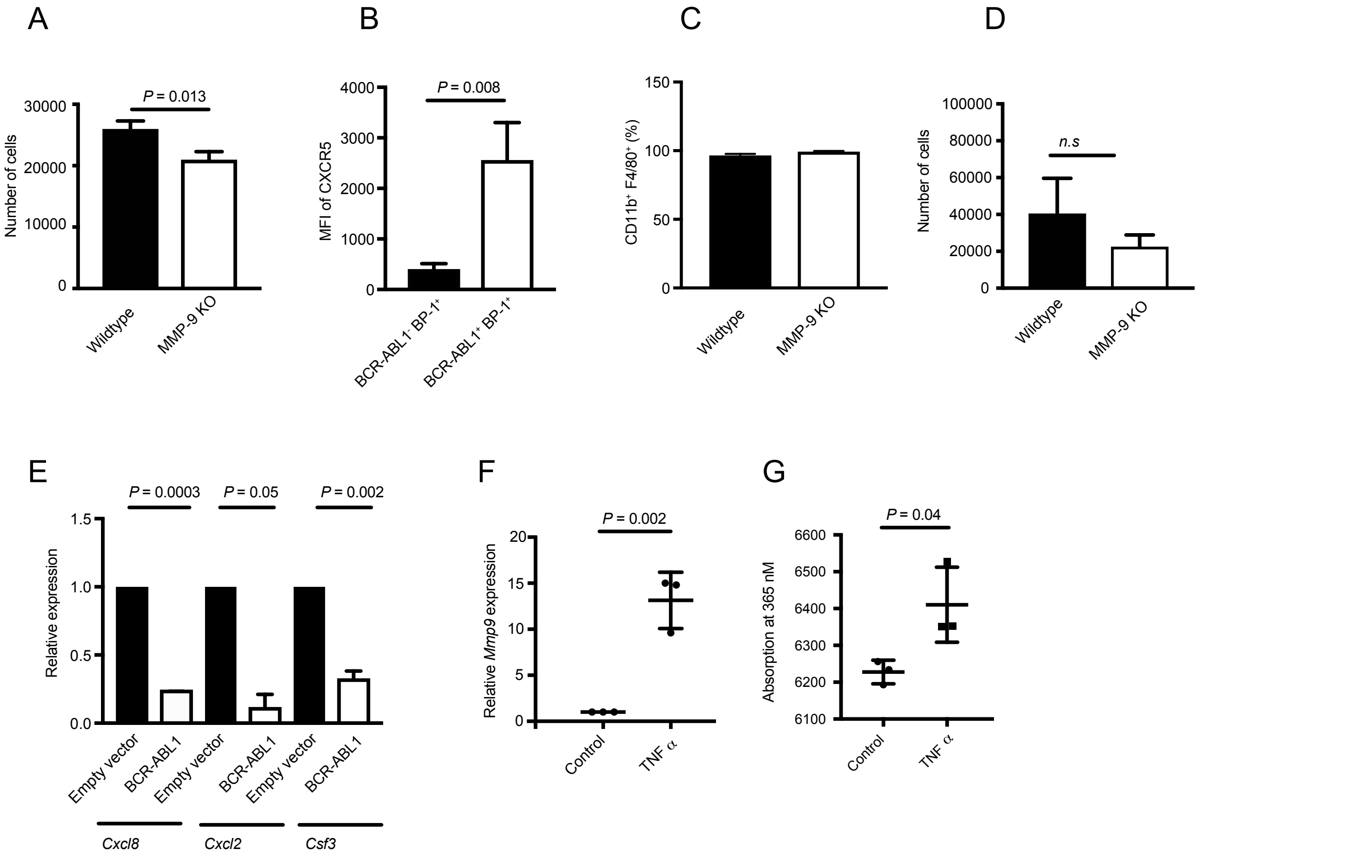

Supplementary Figure 6: A) Number of primary murine BCR-ABL1⁺ BP-1⁺ B-ALL cells which migrated from the upper chamber through a layer of wildtype (black) or MMP-9 KO (white) mesenchymal stromal cells to the lower chamber in a transwell/invasion assay containing 500 ng/ml CXCL13 (*P* = 0.013; t-test, n=4-6). 10^5 leukemia cells had been plated and were allowed to migrate for 24 hours. B) Mean fluorescence intensity (MFI) of CXCR5 on normal, BCR-ABL1 negative BP-1⁺ (black) and BCR-ABL1 positive BP-1⁺ (white) B cells. C) Percentage of CD11b⁺ F4/80⁺ macrophages isolated from bone marrow of wildtype (black) and MMP-9 KO mice (white) after 10 days of culture (n=2). D) Number of primary sorted murine BCR-ABL1⁺ BP-1⁺ B-ALL cells which migrated from the upper chamber through a layer of wildtype (black) or MMP-9 KO (white) macrophages to the lower chamber in a transwell/invasion assay containing 0.5 ng/ml SDF-1 alpha (t-test, n=3). Cells were allowed to migrate for 48 hours. E) Relative expression of *Cxcl8* (*IL-8), Cxcl2 (Gro-beta)* or *Csf3 (G-csf)* in total bone marrow cells from mice transplanted with bone marrow transduced with MSCV-IRES GFP (empty vector) or BCR-ABL1 GFP (t-test, n=3). F) Relative expression of *Mmp9* in MC3T3 cells *in vitro* treated with vehicle (PBS) or 15 ng/ml TNFα for 6 hours (*P* = 0.002; t-test, n=4-5). G) Absorbance (at 365 nm) of fluorescent, emitted light after cleavage of an MMP-9-specific fluorogenic substrate, which had been incubated with the conditioned medium from MC3T3 cells, that were treated with vehicle or 15 ng/ml TNFα (*P* = 0.04; t-test, n=4-5). The conditioned medium had been harvested 48 hours after initiating TNFα treatment, and the substrate had been incubated with the conditioned medium for 30 minutes.

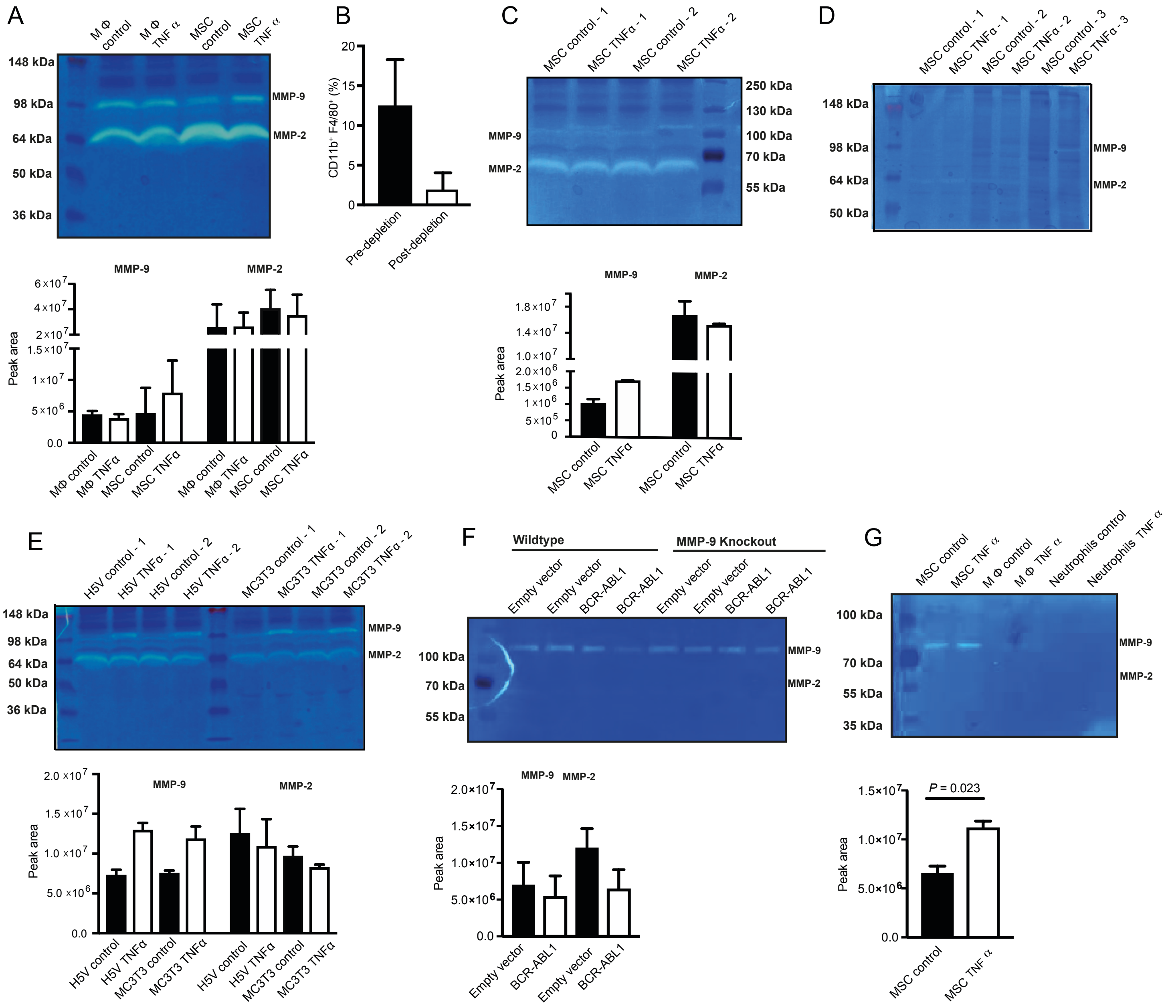

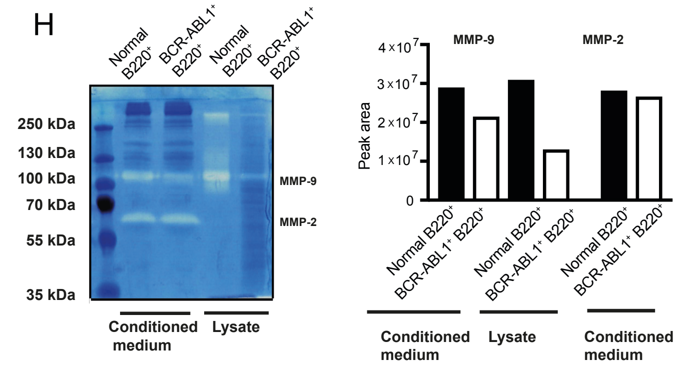

Supplementary Figure 7: A) Gel zymography of the enzymatic levels of MMP-9 and MMP-2 in the conditioned medium (containing serum) of macrophages or MSC treated with vehicle or 15 ng/ml TNFα. The conditioned medium had been harvested 48 hours after initiating TNFα treatment. B) Percentage of $CD11b^+$ $F4/80^+$ macrophages after depletion from MSC. C) Gel zymography of conditioned medium (containing serum) of MSC depleted of $CD11b^+$ $F4/80^+$ cells as in (B). D-E) Gel zymography of the enzymatic levels of MMP-9 and MMP-2 in lysates of levels of MMP-9 and MMP-2 in lysates of MSC (D), in conditioned medium of H5V and MC3T3 cells (E) treated with vehicle or 15 ng/ml TNFα. The conditioned medium (containing serum) had been harvested 48 hours after initiating TNFα treatment. F) Gel zymography of the enzymatic levels of MMP-9 (and MMP-2) in bone marrow fluids of mice transplanted with bone marrow which had been transduced with MSCV-IRES GFP (empty vector) or BCR-ABL1 on day 23 after transplantation. G) Gel zymography of the enzymatic levels of MMP-9 (and MMP-2) in the serum-free conditioned medium of macrophages, neutrophils or MSC treated with vehicle or 15 ng/ml TNFα. The conditioned medium had been harvested 24 hours after initiating TNFα treatment. H) Gel zymography of the enzymatic levels of MMP-9 and MMP-2 in conditioned medium (containing serum) or lysates of sorted $B220^+$ B cells from healthy mice or BCR-ABL1$^+$ $B220^+$ B cells from mice with B-ALL. The amount of conditioned medium loaded in the zymographies in A, C, E, G and H was equal in volume between the different samples and represented the conditioned medium collected from 50,000 cells, i.e. MSC, macrophages, neutrophils, MC3T3 or H5V cells.

Supplementary figure 8

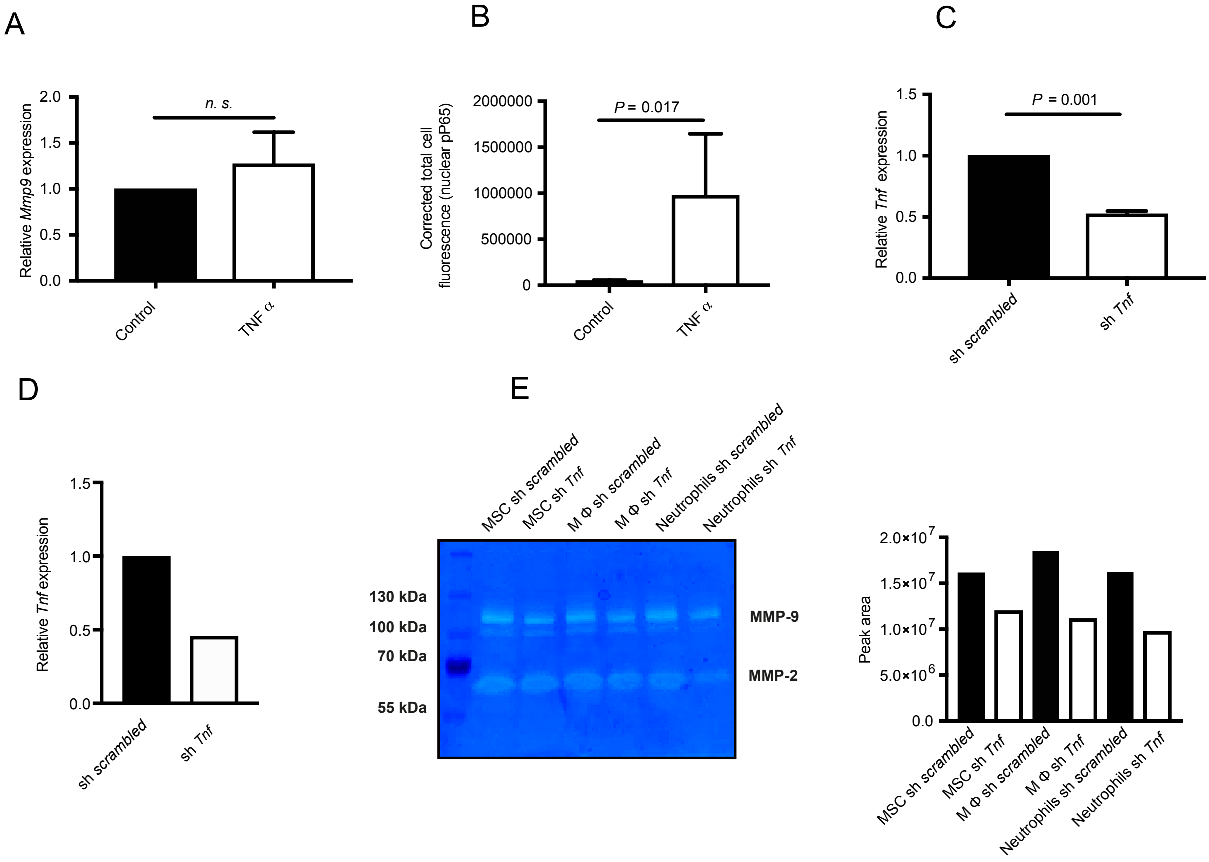

Supplementary Figure 8: A) Relative expression of *Mmp9* in TNF receptor 1 KO mesenchymal stromal cells, which were treated *in vitro* with vehicle (PBS) (black) or 15 ng/ml TNFα for 6 hours (white) (t-test, n=4-5). B) Quantification of the corrected total cell fluorescence of nuclear phospho P65 in mesenchymal stromal cells after 8 hours of treatment with vehicle (black) or 15 ng/ml TNFα (white) (*P* = 0.017; t-test, n=5). C) Relative expression of *Tnf* in 3T3 cells transduced with *scrambled* (black)- or *Tnf* (white)- shRNA-expressing lentivirus (*P* = 0.001; t-test, n=3). D) Relative expression of *Tnf* in BCR-ABL1$^+$ BaF3 cells transduced with sh *scrambled* or sh *Tnf*-expressing lentivirus (t-test,n=2). E) Gel zymography of the enzymatic levels of MMP-9 and MMP-2 in the conditioned medium of MSC, macrophages and neutrophils exposed for 24 hours to the conditioned medium from BCR-ABL1$^+$ BaF3 cells co-transduced with sh *scrambled* or sh *Tnf*-expressing lentivirus. The BCR-ABL1$^+$ BaF3 cells transduced with sh *scrambled*- or sh *Tnf*-expressing lentivirus had been selected for GFP expression.

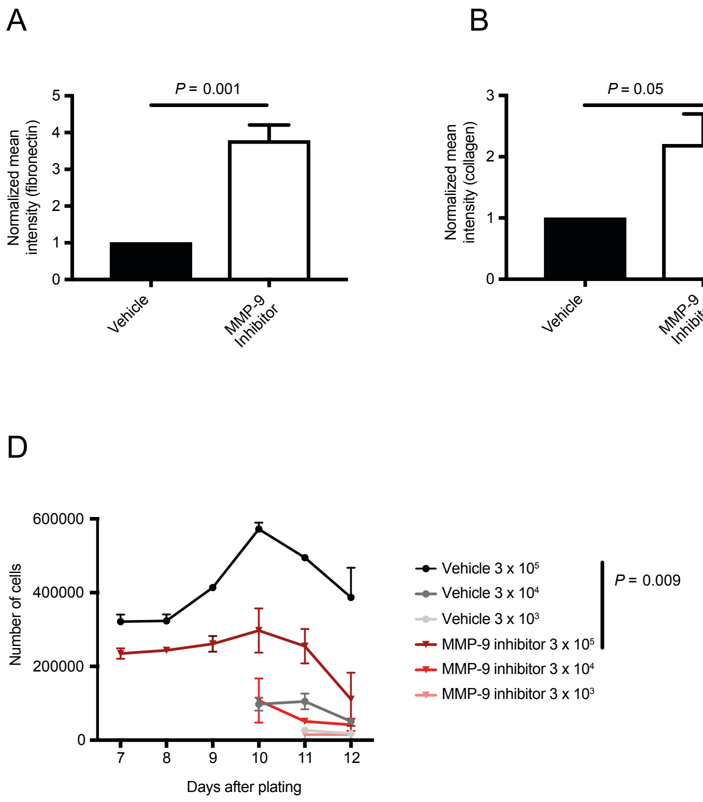

Supplementary Figure 9: A-C) Normalized mean intensity of the extracellular matrix proteins fibronectin (*P* = 0.001; t-test) (A), collagen (*P* = 0.05; t-test) (B) and laminin (*P* = 0.002; t-test) (C) in bone sections of mice with B-ALL treated with vehicle (black) or the MMP-9 inhibitor (white) (20 mg/kg) at the time of death (n=3-4). Treatment was started on day 12 after transplantation and administered 2-3 times/week. The quantification was performed using the Fiji imaging tool. D) Number of cells growing in wells of a 24-well plate after plating of 3 x 10^5, 3 x 10^4 or 3 x 10^3 total bone marrow cells from mice with established B-ALL (day 20 after transplantation) in duplicate.

Supplementary figure 10

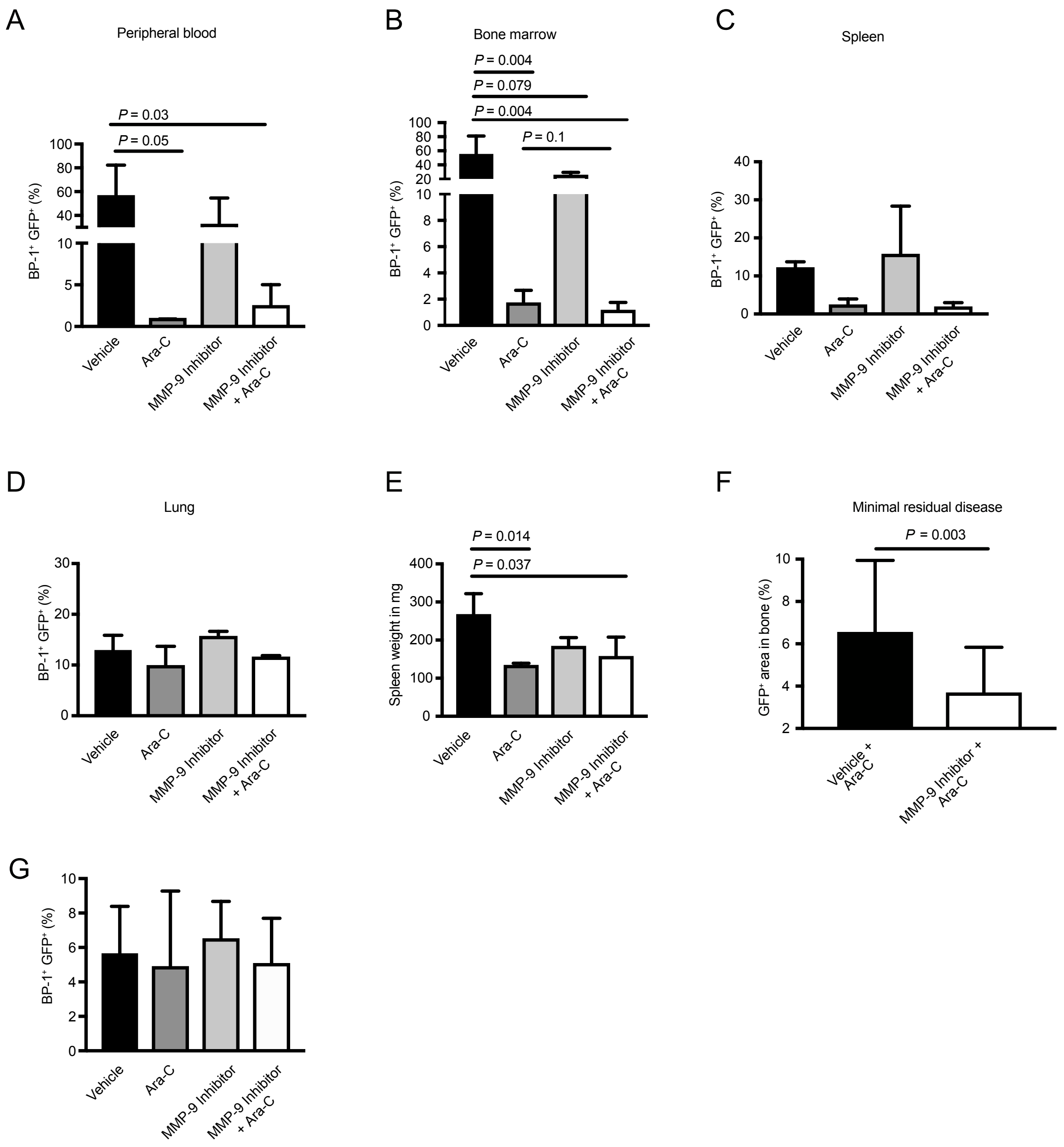

Supplementary Figure 10: A-D) Percentage of GFP$^+$ (BCR-ABL1$^+$) BP-1$^+$ cells in peripheral blood (A), bone marrow (B), spleen (C) and lung (D) of mice transplanted with BCR-ABL1-transduced bone marrow in the B-ALL model treated with vehicle (black), ara-C (dark gray), MMP-9 inhibitor (light gray) and MMP-9 inhibitor plus ara-C (white) on day 25 after transplantation (n=3). E) Spleen weights of mice as in (A-D) on day 25 after transplantation (One-way ANOVA; Tukey Test; n=3). F) Percentage of the GFP$^+$ area (representing BCR-ABL1$^+$) cells (detected by immunoperoxidase using yellow-brown horse-radish-peroxidase chromogen) of the entire area in bone sections of mice with B-ALL co-treated with ara-C and vehicle (black) or ara-C and the MMP-9 inhibitor (white) on day 54 after transplantation (*P* = 0.003, t-test, n=4-5). G) Percentage of GFP$^+$ (BCR-ABL1$^+$) BP-1$^+$ cells in peripheral blood of mice transplanted with BCR-ABL1-transduced bone marrow in the B-ALL model on day 12 after transplantation, i.e. before the start of treatment (One-way ANOVA; Tukey Test).

Supplementary figure 11

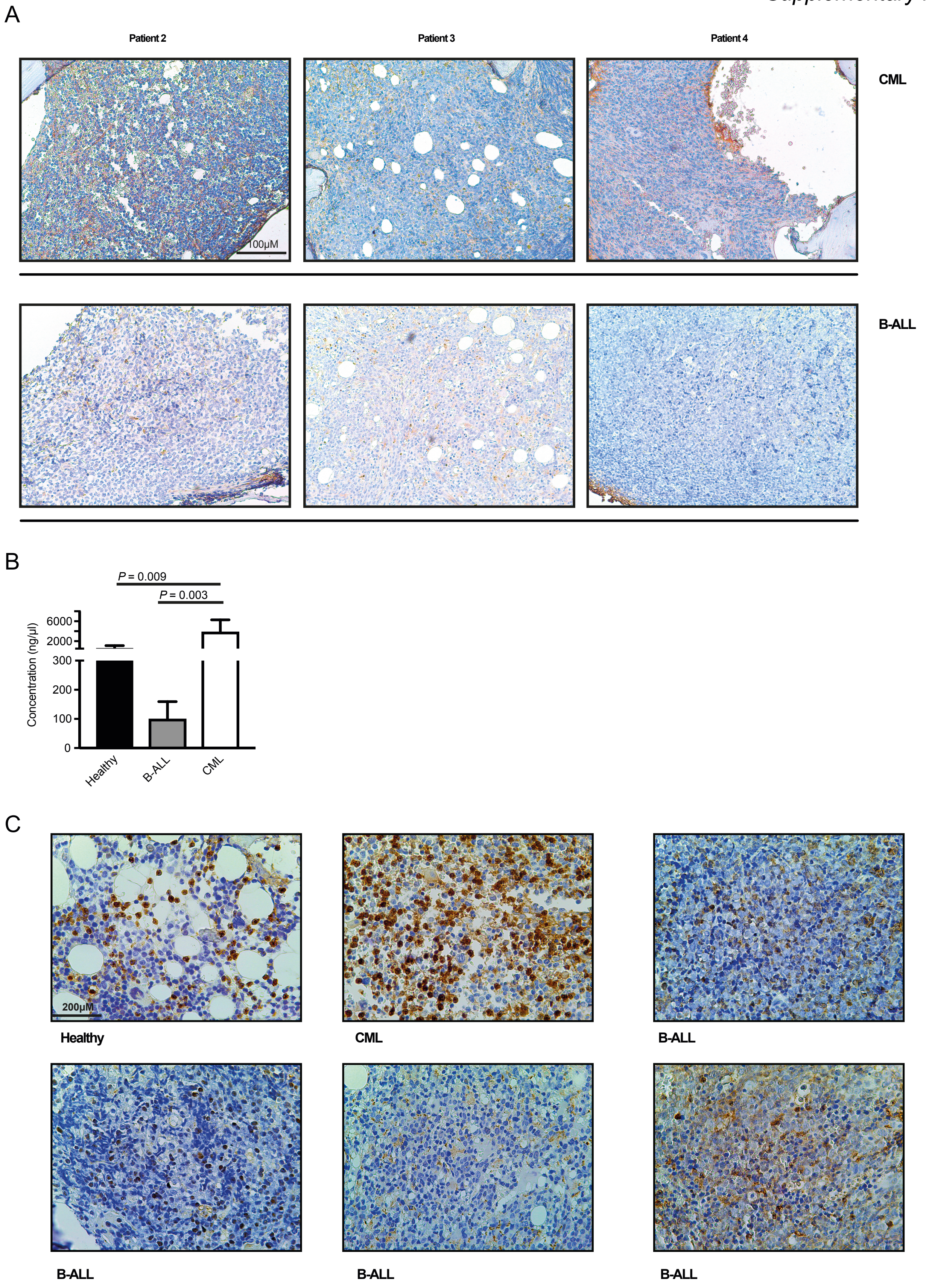

Supplementary Figure 11: A) Representative immunohistochemistry images of bone sections of 3 human patients with chronic myeloid leukemia (CML; top) or 3 patients with B-cell acute lymphoblastic leukemia (B-ALL; bottom) stained with an antibody to fibronectin (detected by immunoperoxidase using yellow-brown horseradish-peroxidase chromogen). The scale bar depicts 100 µm. B) Concentration in ng/µl of human MMP-9 in the plasma of bone marrow aspirates of healthy controls (black), patients with B-ALL (gray) and patients with CML (white) (One-way ANOVA; Tukey test; n=4-8). C) Representative immunohistochemistry images of bone sections of a healthy human control, a human patient with CML and four patients with B-ALL stained with an antibody to human MMP-9 (detected by immunoperoxidase using yellow-brown horseradish-peroxidase chromogen). The scale bar depicts 200 µM.

Table S1: Antibodies used in the study

Antibodies used for flow cytometry

Antibody	Fluorochrome	Clone	Company	Catalogue number
Streptavidin	APC-Cy7		Biolegend, San Diego, CA	405208
Gr-1	Biotin	RB6-8C5	BD Biosciences, San Jose, CA	663125
Gr-1	APC-Cy7	RB6-8C5	BD Biosciences, San José, CA	557661
BP-1	Biotin	6C3	Biolegend, San Diego, CA	108304
Annexin V	PE		Life technologies, California, USA	A35111
Ki-67	PE	16A8	Biolegend, San Diego, CA	652404
CD48	PB	HM48-1	Biolegend	103413
CD150	PE	Q38-480	BD Biosciences, San Jose, CA	562651
Sca-1	PE-Cy7	D7	Biolegend, San Diego	108114
c-Kit	APC	2B8	BD Biosciences, San Jose, CA	553356
CD5	APC-Cy7	53-73	BD Biosciences, San Jose, CA	553019
B220	PB	RA3-6B2	BD Biosciences, San Jose, CA	553086
F4/80-Biotin		BM8	eBiosciences, San Diego	13-4801-82
Ly6G & Ly6C-Biotin		RB6-8C5	BD Biosciences, San Jose, CA	553125
Ter-119	FITC	RE A847	BD Biosciences, San Jose, CA	553672
B220	PE	RA3-6B2	Biolegend, San Diego	103227
CD11b	APC	M1/70	BD Biosciences, San Jose, CA	553312
CD45	PE	30-F11	BD Biosciences, San Jose, CA	553081
CXCR5	APC	2G8	BD Biosciences, San Jose, CA	560615
CD31	FITC	MEC133	BD Biosciences, San Jose, CA	553372
CD16/32	BV450	2.4G2	BD Biosciences, San Jose, CA	560539
CD34	PE	RAM34	BD Biosciences, San Jose, CA	551387
CD127	BV711	BV711	BD Biosciences, San Jose, CA	135035
CD29	FITC	FITC	Biolegend, San Diego, CA	102206
CD90	APC	OX7	BD Biosciences, San Jose, CA	563772
CD44	PB	IM7	Biolegend, San Diego, CA	103020
CD105	PerCF594	M57/18	BD Biosciences, San Jose, CA	562762
CD49e	BV650	HM alpha 5-1	BD Biosciences, San Jose, CA	743112
PDGFR alpha	APC	APA 5	Biolegend, San Diego, CA	17-1401-81

PE=Phycoerythrin; APC= Allophycocyanine; Cy=Cyanine

Antibodies used for immunohistochemistry and immunofluorescence

**Antibody**	**Clone**	**Company**	**Catalogue number**
pP65		Cell Signaling, Danvers, Massachusetts	3033
P65		Merck, Darmstadt, Germany	I7-10060
GFP		Abcam, Cambridge, UK	ab6673
Collagen		Abcam, Cambridge, UK	ab6586
Laminin		Abcam, Cambridge, UK	ab11575
Fibronectin		Abcam, Cambridge, UK	ab2413
Lamin B1		Abcam, Cambridge, UK	ab16048
Rabbit HRP		Cell Signaling, Danvers	70748
Goat HRP		Abcam, Cambridge, UK	ab97110
Human MMP-9	5G3	Abcam, Cambridge, UK	ab119906

Table S2: Primers used in the study

Genes	Forward primer	Reverse primer
Vegfa	GGAGAGCAGAAGTCCCATGA	ACACAGGACGGCTTGAAGAT
Tnf	GCCTATGTCTCAGCCTCTTCT	AACTGATGAGAGGGAGGCCATT
Mmp9	TCCAGTTTGGTGTCGCGG	AAGGGGAAGACGCACAGC
Mmp9 CHIP set 1	GCGACAAAGGGTCTGTTTTTG	TGACTCAGCTTCCTCTCCTGG
Mmp9 CHIP set 2	GCCTGCTGGAGCTAGGGGTTTG	GAGTGCAGCCTGGAGCCCATC
Il1a	AACGTCAAGCAACGGGAAGA	GCTGATCTGGGTTGGATGGT
Il1b	AATTGGTCATAGCCCGCACT	AAGCAATGTGCTGGTGCTTC
Il6	AGCCAGAGTCCTTCAGAGAGAT	AGGAGAGCATTGGAAATTGGGG
Gapdh	AGGTCGGTGTGAACGGATTTG	GGGGTCGTTGATGGCAACA
Cxcl8 (IL-8)	CAGGCCTGACATCTTCTGCAA	CTGACATGGCAGCCATTGT
Cxcl2 (Gro-beta)	GAGCTTGAGTGTGACGCCCCCAGG	GTTAGCCTTGCCTTTGTTCAGTATC
Csf3 (G-csf)	CGAAGGCTTCCCTGAGTGGCTGCTCTA	GGACACCTCCTGCCCGGCGCTGG

Gapdh=glyceraldehyde 3-phosphate dehydrogenase
Vegfa=Vascular endothelial growth factor A; Tnf=Tumor necrosis factor α; Mmp9=Matrix metallopeptidase-9; Il1a=Interleukin 1 alpha; Il1b=Interleukin 1 beta; Il6=Interleukin 6; Cxcl8 (IL-8)=Interleukin 8; Cxcl2=Gro-beta; Csf3 (G-csf)=granulocyte-colony stimulating factor

Supplementary materials and methods

Drug treatment

Mice were treated two to three times a week with vehicle (corn oil) or the selective MMP-9 inhibitor II (Cat. no. 444293, Merck, Darmstadt, Germany)[1] by intraperitoneal injection at 20mg/kg/dose starting from day 12 after transplantation [2].

Cytarabine (ara-C) was administered via intraperitoneal injection once a day at 50mg/kg (Sigma-Aldrich, Munich, Germany) for 5 days starting from day 12 of transplantation. Treatment was then paused for 2 weeks, and, consequently, reinitiated for 5 days.

Bone marrow transduction and transplantation

Generation of MSCV IRES GFP BCR-ABL1-expressing retrovirus and transplantation were performed as described [3]. Briefly, donor bone marrow (BM) cells were harvested from wildtype mice, transduced with cryopreserved retrovirus expressing MSCV-IRES GFP BCR-ABL1 (p210) and intravenously transplanted into sublethally irradiated recipient mice (900 cGy) at a dose of 1×10^6 cells.

Secondary transplantation was performed by injecting $2\text{-}3\times10^6$ cells from primary mice with established B-ALL (day 20 after transplantation) into sublethally irradiated wildtype secondary recipients.

In the homing assay, 5×10^6 wildtype BM cells, transduced with BCR-ABL1 were intravenously injected into C57/BL6 and MMP-9 deficient mice. 18 hours later BM and spleen of the recipient mice were analysed for GFP^+ ($BCR\text{-}ABL1^+$) $BP1^+$ cells.

For the *Tnf* shRNA knockdown experiment, bone marrow cells were co-transduced with MSCV IRES RFP BCR-ABL1 retrovirus and *scrambled* or *Tnf* shRNA-expressing lentivirus. Lentivirus was generated via transfection of 293T cells with the *scrambled* or *Tnf* shRNA GIPZ vectors, Delta 8.9 and VSV, as described [3].

Analysis of diseased mice and tumor burden

The leukemic burden in diseased animals was assessed by analysis of peripheral blood by a Scil Vet animal blood counter (Scil Animal Care Company, Viernheim, Germany) and by staining leukocytes from peripheral blood with a phycoerythrin (APC-Cy7)-conjugated antibody to BP-1 (Biolegend, San

Diego, CA). GFP and BP-1 expression allowed enumeration of leukemia cells.

To test infiltration of different organs by GFP^{+} (BCR-ABL1^{+}) BP1^{+} leukemia cells, peripheral blood, bone marrow, spleen and lung were harvested from mice with B-ALL treated with vehicle, ara-C, the MMP-9 inhibitor or ara-C plus the MMP-9 inhibitor on day 20 after transplantation. Organs were crushed in PBS supplemented with 2% fetal calf serum. Subsequently, the cells were stained for BP-1 and processed for flow cytometry.

In order to create a hematological profile of healthy wildtype and MMP-9 knockout mice, bones and spleen were crushed in PBS containing 2% FBS and cells stained for differentiated cells, lineage (CD5, CD11b, Ter119, B220), c-Kit, Sca-1 (LKS) cells, LKS CD150^{+} CD48^{-} (SLAM) cells, myeloid progenitors (multipotent progenitors (MPP): Lin$^{-/lo}$ c-Kit^{high} Sca-1^{hi} Thy1.1^{-} Flk2^{+}, common myeloid progenitors (CMP): IL-7Rα^{-} Lin^{-} Sca-1^{-} c-Kit^{+} FcγR^{lo} CD34^{+}), megakaryocyte erythroid progenitors (MEP): IL-7Rα^{-} Lin^{-} Sca-1^{-} c-Kit^{+} FcγR^{lo} CD34^{-}, granulocyte-monocyte progenitors (GMP): IL-7Rα^{-} Lin^{-} Sca-1^{-} c-Kit^{+} FcγR^{hi} CD34^{+}, as described [4].

Conditioned medium and quantification of TNFα

Conditioned medium was harvested after 6-7 days of culturing MSC. Bone marrow supernatant was harvested by flushing the long bones of control or leukemic mice with 250 µl PBS. TNFα was detected in the conditioned medium or bone marrow supernatant using an enzyme-linked immunosorbent assay (ELISA) (Cat. no. 430901, Biolegend, Koblenz, Germany) according to the manufacturer's instructions.

Isolation of mesenchymal stromal cells

Mesenchymal stem cells were obtained by cutting bones in small pieces and digesting them in collagenase (Sigma, Munich, Germany) for 1 hour at 37 °C. CD45- Ter119- PDGFRα^{+} Sca1^{+} MSC were sorted, as described [5], and then cultured in α-MEM medium supplemented with 20% fetal bovine serum, 1% penicillin/streptomycin, 1% L-glutamine and primocin (InvivoGen, Toulouse, France). The immunophenotype of MSC was confirmed by staining for CD45, CD34, Ter119, CD11b, F4/80, CD31, CD49e, CD90, CD29, CD44, CD105, Sca1 and PDGFRα and flow cytometry. MSC were used prior to passage six.

In indicated experiments, MSC were depleted of macrophages using anti-CD11b and -F4/80-biotinylated antibodies and streptavidin microbeads by MACS columns. MSC were cultured in α-MEM with 20 % fetal bovine serum, 1% L-glutamine and and 1% penicillin/streptomycin.

In-vitro assays

30,000 mesenchymal stromal cells were plated on top of the transwell (5 µm size; Sarstedt, Nürnbrecht, Germany) 24 hours prior to the start of the invasion assay. Consequently, 80,000 sorted GFP+ (BCR-ABL1+) B220+ cells from mice with established B-ALL were added on top of the MSC or macrophages. After 48 hours B-ALL cells which had migrated towards the lower chamber of the transwell, which contained 0.5 ng/ml of the chemoattractant stromal derived factor (SDF)-1 (Peprotech, Hamburg, Germany), were enumerated.

For the co-culture assay, 50,000 mesenchymal stromal cells were cultured in a 12-well plate. Once the MSC were confluent, the cells from the bone marrow of mice with established B-ALL were added on top. After 48 hours of coculture the leukemia cells were removed and the coculture was washed briefly three times using PBS. MSCs were collected by trypsinization and the cells were processed further for RNA isolation.

50,000 MC3T3 cells were cultured in α-MEM medium supplemented with 1% L-glutamine and 20% FBS on day -1. Cells were treated with 15 ng/ml TNFα for 6 hours. After the treatment cells were trypsinized, collected and processed further for qPCR.

In the B cell plating assay, total bone marrow cells from mice with established B-ALL (day 20 after transplantation) were plated in triplicate in limiting dilution, i.e. 3 x 10^5, 3 x 10^4 and 3 x 10^3 on MSC, which had been plated at 10,000 cells/well in a 24-well plate. The leukemia cells were counted daily beginning on day 7 after plating.

For zymography studies, 50,000 neutrophils, MSC, macrophages, H5V or MC3T3 were cultured in a well of a 12-well plate. Once the cells reached 80 % confluence, 15 ng/ml of TNF-α or conditioned medium from BCR-ABL1+ (red fluorescent protein; RFP+) BaF3 cells, which had been transduced with sh *scrambled* or sh Tnf-expressing lentivirus, as described above, were added to the cultures. After 24 or 48 hours, as indicated in the legends, the conditioned medium or cells were collected for zymography.

Lentivirally transduced cells were selected by daily addition of 1 μg/ml puromycin for 10 days. At the time of the experiment the cells were 85% GFP+ and 100% RFP+.

In the methylcellulose assays, 10 × 10^4 total bone marrow cells from WT or MMP-9 knockout mice were plated in methylcellulose in triplicate (M3434, Stem Cell Technologies, Vancouver, Canada). After 7 and 10 days colonies were scored.

Differentiation of MSC

15,000 MSC were seeded in a well of a 48-well plate. MSC were differentiated into adipocytes using differentiation and maintenance media (Cat. No. PT3102A, PT3102B, LONZA, Cologne, Germany) and stained with oil red. MSC were differentiated into osteoblasts using α-MEM with 20 % FCS supplemented with 50 μg/ml ascorbic acid, 10 mM β-glycerophosphate and 10 mM dexamethasone for 10 days [6]. The differentiated cells were fixed and stained by Von Kossa.

Isolation of bone marrow macrophages and neutrophils

Bones of wildtype versus MMP-9 knockout mice were crushed and the crushed bones were plated in a tissue culture dish in α-MEM medium supplemented with 20% fetal bovine serum, 1% penicillin/streptomycin and 1% L-glutamine, as described [7]. Non-adherent cells were removed after 7 days. Approximately 14 days after plating (or when the macrophages were 70-80 % confluent) the adherent cells were immunophenotyped for macrophage markers and used in experiments. Neutrophils were purified from flushed bone marrow via staining with a biotinylated antibody to Gr-1 (BD Biosciences, San José, CA, USA) and streptavidin-coupled microbeads and magnetic-activated cell sorting (MACS) (Miltenyi, Bergisch Gladbach, Germany). Purified neutrophils were maintained in RPMI 1640 supplemented with 20 % fetal bovine serum, 1% L-glutamine and 1% penicillin/streptomycin for 24 hours.

Immunofluorescence studies

MSC were cultured on poly-L-lysine-coated cover slips. MSC were treated with 15 ng/ml TNFα for 8 hours at 37°C. The cells were then fixed with 4 % paraformaldehyde for 20 minutes at room temperature

and permeabilized with 0.25 % Triton X-100 for 10 minutes. The cells were then blocked with 2% BSA and incubated with a primary antibody to p-P65 (Cell Signaling, Danvers) at a 1:50 dilution overnight at 4 °C. Cells were washed and incubated with the secondary antibody Alexa Fluor 647 (Invitrogen, Grand Island, NY) at a 1:500 dilution for one hour at room temperature. The slides were mounted using mounting media with DAPCO (Cat. no. 290734; Sigma-Aldrich, Munich, Germany), and images were taken on a confocal laser scanning microscope (CSLM) (Leica, Wetzlar, Germany). Quantification of nuclear p-P65 was performed using ImageJ. The p-P65 signal was corrected by subtraction of background signal. The integrated density of nuclear p-P65 was calculated with the formula CTCF = Integrated Density – (Area of selected cell x mean fluorescence of background readings).

Immunofluorescence of bone sections

Bones were collected from mice and fixed in 4% paraformaldehyde overnight at 4°C. Decalcification was performed with 0.5 M EDTA for one week. Bones were rehydrated with 2% polyvinylpyrrolidone (PVP) overnight at 4°C. Consequently, bones were frozen in OCT and stored at -80°C. Immunofluorescence staining was performed after bone sectioning, as described above. Permeabilization with 0.3% Triton X-100 was followed by incubation in 3% BSA at room temperature for 30 minutes and staining with primary antibodies for fibronectin, collagen and laminin (all antibodies were from Abcam, Cambridge, UK). Primary antibodies were diluted to 1:200 and incubated on the bone sections overnight. Alexa Fluor-647 (Thermo Fisher Scientific, Waltham, MA)-coupled anti-rabbit secondary antibody (1:400) was used for 1 hour at room temperature. DAPI (Merck, Darmstadt, Germany) was used to stain nuclei at a 1:2000 dilution. Subsequently, bones were briefly washed with PBS and mounted with FluorMount plus 50 µg/ml DABCO (Sigma-Aldrich, Munich, Germany). Images were acquired with a CQ1 confocal microscope (Yokogawa, Amersfoort, The Netherlands) using the same settings.

Immunohistochemistry

Tissue samples of murine meninges or bone sections from a healthy individual or patients with B-ALL or CML were processed, embedded, sectioned and subjected to immunohistochemistry analysis for GFP

(ab6673, Abcam, Cambridge, UK) fibronectin (ab2413, Abcam, Cambridge, UK) or MMP-9 (ab119906, Abcam, Cambridge, UK), respectively, following standard procedures. Bones were decalcified in 0. 5M EDTA.

Flow cytometric analysis of cell cycle status and apoptosis

For the evaluation of apoptosis, cells were stained with Annexin V (Invitrogen, Grand Island, NY) and DAPI. Analysis of cell cycle was performed by fixing cells and staining overnight with Ki-67 (Cat no. 652403; Biolegend, San Diego) according to the manufacturer's instructions. DAPI was added 30 minutes prior to flow cytometric analysis. The stained cells were acquired on a BD LSR Fortessa (BD Biosciences, Franklin Lakes, NJ).

Western blotting

Bones were collected from mice, and the bone marrow was flushed with PBS. The rest of the bones was crushed and pelleted by centrifugation. The pellet was then resuspended in RIPA buffer (50 mM Tris HCL, pH 7.4, 150 mM NaCl, 1% Triton X-100, 1% NaDOC, 0.1% SDS and 1 mM EDTA, 50 mM NaF, 1 mM Na_3VO_4, aprotinin and PMSF), to which protease and PhosSTOP phosphatase inhibitors (Cat. no. 4906845001, Roche, Basel, Switzerland) had been added, and incubated on ice for 30 minutes. Cells were centrifuged at 4°C at 14000 rpm for 10 min. The collected supernatant represents the protein fraction that was used for further analysis. Protein concentrations of RIPA lysates were measured using the Bradford protein assay (Bio-Rad, Hercules, CA). Equal concentrations of samples were mixed with the SDS running buffer Roti-load 4X (Cat no. K929.1; Carl Roth, Karlsruhe, Germany), boiled at 95°C for 5 minutes and loaded on precast SDS-PAGE gels (Thermo Fisher Scientific, Darmstadt, Germany). The proteins were then transferred onto a nitrocellulose membrane (Cat. no. 88018, Thermo Fisher Scientific, Darmstadt) and probed with antibodies to fibronectin (Cat. no. Ab2413, Abcam, Cambridge, UK) or the nuclear membrane marker lamin B1 (Cat. no. Ab16048, Abcam, Cambridge, UK). 5% milk powder in TBST was used for blocking. Anti-rabbit-HRP (Cat. no. 70748, Cell Signaling, Danvers) and anti-goat-HRP (Cat. no. ab97110, Abcam, Cambridge, UK) were used as secondary antibodies.

Quantitative PCR

Quantitative PCR was performed using standard protocols, and primers are listed in Table S2.

ChIP Assay

After treating MSC with 15ng/ml TNFα for 6 hours cells were harvested by trypsinization and were cross-linked with formaldehyde, and the reaction was quenched using glycine, as described [8]. After washing in cold PBS, the pellet was resuspended in ChIP lysis buffer (50mM HEPES KOH, 140 mM NaCl, 1% Triton X-100, imM EDTA pH 8, 0.1% Sodium deoxycholate, 0.1% SDS). After 1 hour of lysis at 4°C lysates were sonicated to shear DNA to an average fragment length of 500-1000 bp. The cell debris was pelleted and the supernatant was harvested, aliquoted in new tubes, snap-frozen in liquid nitrogen and finally stored at -80°C for further analysis. For immunoprecipitation, blocked protein A beads, ChIP lysates and a P65 antibody (Cat. no. I7-10060, Merck, Darmstadt) (4 µg) were incubated overnight at 4°C. A rabbit IgG antibody was used as control (Cat. no. sc-2027, Santa Cruz Biotechnology, Dallas). After several wash steps, DNA was eluted and proteinase K was added. Finally, the ChIP DNA was purified using the ChIP DNA Clean and Concentrator kit (Zymo Research, Irvine, USA). The DNA was eluted with 40 µl of the elution buffer, and qPCR analysis was performed using 2 µl of chromatin with two different primer sets specific for regions, at which P65 binds within the *Mmp9* promoter [9, 10]. Primers are listed in Table S2. DNA recovery was calculated as percent of the input.

Degradation assay

100 µl of conditioned medium was mixed with 100 µl of 2x assay buffer (400nM NaCl, 100mM Tris-HCL pH 7.6, 10mM Cacl2, 40µM $ZnSO_4$, 0.1% Brij 35) and incubated for 30-45 minutes with 10 µM fluorogenic substrate (Cat. no. 444221; Calbiochem, Darmstadt) at 37° C in the dark. Absorption of fluorescence, emitted due to MMP-9-dependent cleavage of fluorogenic substrate was measured at 365 nm.

Enzyme-linked immunosorbent assay

Human bone marrow plasma samples were obtained from 5 healthy donors, 5 B-ALL and 4 CML

patients at diagnosis. The concentration of human MMP-9 was determined using the Human MMP-9 platinum ELISA kit (BMS2016/2TEN, Thermo Fisher Scientific, Waltham, USA) according to the manufacturer´s instructions. The optical density (OD) was measured by a plate reader (Spectra Max 340, MWG Biotech, Ebersburg, Germany) at 450 nm. A polynomial curve analysis was performed to generate a standard curve from the average absorbance of standards. Human MMP-9 concentrations were calculated by fitting the average of duplicate ODs of each sample with the standard curve.

Studies were approved by local ethics committee bylaws in the hematology departments (Hospital Lyon Sud, Pierre Bénite and Centre Leon Bérard, Lyon, France) involved in this study. For CML patients' samples, definitions of response respected the last European Leukemia Net Recommendations.

All donors provided written informed consent in accordance with the Declaration of Helsinki.

Zymography

Conditioned medium in serum-free or serum-containing condition, as indicated in the legends, or lysates, prepared in RIPA buffer, from neutrophils, macrophages, MSC, H5V or MC3T3 cells untreated or treated with TNFα (15 ng/ml) or conditioned medium from BCR-ABL1^{+} BaF3 cells transduced with sh *scrambled*- or sh *Tnf*-expressing lentivirus were harvested after 24 or 48 hours, as indicated in the legends. In the case of the BCR-ABL1^{+} (red fluorescent protein; RFP^{+}) BaF3 cells transduced with sh *scrambled-* or sh *Tnf*-expressing (GFP+) lentivirus the cells were selected, as described above. Their conditioned medium was harvested, and MSC, neutrophils or macrophages cultured in this conditioned medium, before their conditioned medium was used for the zymography studies. Total bone marrow fluid was isolated by flushing one femur from healthy or leukemic wildtype or MMP-9 KO mice.

Cell lysates were quantified using the BCA colorimetric quantification method (Cat.No. 5000006, Bio-Rad, Feldkirchen, Germany). The conditioned medium was equal in volume (25 μl) between the different samples and represented the conditioned medium collected from 50.000 cells, i.e. MSC, macrophages, neutrophils, MC3T3 or H5V cells. This conditioned medium was mixed with 4 x Laemmli Buffer (without β-mercaptoethanol), left at room temperature for 10 min and then loaded on zymography gels containing 1% gelatin B, as described [11]. For lysates, 5-30 μg of protein lysates mixed with 4 x Laemmli Buffer (without β-mercaptoethanol) were quantified and loaded on zymography gels

containing 1% gelatin B. The zymography gels were prepared as described [11].

References

1. Dufour A, Sampson NS, Li J, Kuscu C, Rizzo RC, Deleon JL*, et al.* Small-molecule anticancer compounds selectively target the hemopexin domain of matrix metalloproteinase-9. *Cancer Res* 2012; **71**(14)**:** 4977-4988.

2. Dufour A, Sampson NS, Li J, Kuscu C, Rizzo RC, Deleon JL*, et al.* Small-molecule anticancer compounds selectively target the hemopexin domain of matrix metalloproteinase-9. *Cancer Res* 2011; **71**(14)**:** 4977-4988.

3. Krause DS, Fulzele K, Catic A, Sun CC, Dombkowski D, Hurley MP*, et al.* Differential regulation of myeloid leukemias by the bone marrow microenvironment. *Nature Medicine* 2013; **19**(11)**:** 1513-1517.

4. Akashi K, Traver D, Miyamoto T, Weissman IL. A clonogenic common myeloid progenitor that gives rise to all myeloid lineages. *Nature* 2000; **404:** 193-197.

5. Houlihan DD, Mabuchi Y, Morikawa S, Niibe K, Araki D, Suzuki S*, et al.* Isolation of mouse mesenchymal stem cells on the basis of expression of Sca-1 and PDGFR-α. *Nat Protoc* 2012; **7**(12)**:** 2103-2111.

6. Mukherjee S, Raje N, Schoonmaker JA, Liu JC, Hideshima T, Wein MN*, et al.* Pharmacologic targeting of a stem/progenitor population in vivo is associated with enhanced bone regeneration in mice. *J Clin Invest* 2008; **118**(2)**:** 491-504.

7. Verma D, Kumar R, Pereira R, Karantanou C, Zanetti C, Minciacchi VR*, et al.* Vitamin K-antagonism impairs the bone marrow microenvironment and hematopoiesis *Blood* 2019; **134**(3)**:** 227-238.

8. Kolodziej S, Kuvardina ON, Oellerich T, Herglotz J, Backert I, Kohrs N*, et al.* PADI4 acts as a coactivator of Tal1 by counteracting repressive histone arginine methylation. *Nat Comm* 2014; **5:** 3995.

9. Kowluru RA, Santos JM, Zhong Q. Sirt1, a negative regulator of matrix metalloproteinase-9 in diabetic retinopathy. *Invest Ophthalmol Vis Sci* 2014; **55**(9)**:** 5653-5360.

10. Qi J, Liu Y, Yang P, Chen T, Liu XZ, Yin Y*, et al.* Heat shock protein 90 inhibition by 17-Dimethylaminoethylamino-17-demethoxygeldanamycin protects blood-brain barrier integrity in cerebral ischemic stroke. *Am J Transl Res* 2015; **7**(10)**:** 1826-1837.

11. Frankowski H, Gu YH, Heo JH, Milner R, Del Zoppo GJ. Use of gel zymography to examine matrix metalloproteinase (gelatinase) expression in brain tissue or in primary glial cultures. *Methods Mol Biol* 2012; **814:** 221-233.
